# The Effects of Concurrent Training on Molecular, Functional, and Clinical Outcomes in Breast Cancer Survivors: A Pilot Study

**DOI:** 10.3390/cancers17121967

**Published:** 2025-06-13

**Authors:** Celia García-Chico, Susana López-Ortiz, Salvador Santiago-Pescador, Paloma Guillén-Rogel, Saúl Peñín-Grandes, Lisa Musso-Daury, Francisco Javier Iruzubieta-Barragán, José Pinto-Fraga, Sergio Maroto-Izquierdo, Lourdes del Río Solá, Alejandro Santos-Lozano

**Affiliations:** 1 i+HeALTH Strategic Research Group, Department of Health Sciences, Miguel de Cervantes European University, 47012 Valladolid, Spain; cgarciac@uemc.es (C.G.-C.); spenin@uemc.es (S.P.-G.); lmusso@uemc.es (L.M.-D.); fjpinto@uemc.es (J.P.-F.); smaroto@uemc.es (S.M.-I.); asantos@uemc.es (A.S.-L.); 2 Department of Health Sciences, Miguel de Cervantes European University, 47012 Valladolid, Spain; ssantiago@uemc.es (S.S.-P.); pguillen@uemc.es (P.G.-R.); fjiruzubieta@uemc.es (F.J.I.-B.); 3 Proporción A, Applied Sports Science Center, 47015 Valladolid, Spain; 4 Vascular Surgery Department, University Hospital of Valladolid, 47003 Valladolid, Spain; mlrio@uemc.es; 5 Physical Activity and Health Research Group (“PaHerg”), Research Institute of Hospital “12 de Octubre” (“imas12”), 28041 Madrid, Spain

**Keywords:** breast neoplasms, breast cancer lymphedema, exercise, inflammation, physical activity

## Abstract

**Background**: Breast cancer survivors (BCS) experience long-term adverse effects, with breast cancer-related lymphedema (BCRL) being one of the most common complications. Exercise is suggested as a safe strategy to improve functionality in BCS with or at risk of developing BCRL. However, the effects of concurrent training in these patients are poorly understood. The aim of the study was to analyze the effects of a 12-week supervised concurrent training program and a 12-week follow-up period without training on molecular, functional, and clinical outcomes in BCS. **Methods**: A single-arm study was conducted in 11 BCS with or at risk of BCRL to analyze the effects of a 12-week concurrent training and a 12-week follow-up period on molecular (92 inflammation-related proteins), functional (upper- and lower-body strength, handgrip strength, and cardiorespiratory fitness), and clinical (body mass index, arm volume, subcutaneous and muscle thickness, range of motion, physical activity levels and heart rate variability, pain, and quality of life [QoL]) outcomes. **Results**: The 12-week concurrent training program significantly improved upper-body muscle strength, handgrip strength, pain, emotional well-being, and total QoL. In addition, after the 12-week follow-up period, the increase in row strength was maintained, and a significant decrease in various inflammation-related proteins was observed. **Conclusions**: A 12-week concurrent training program improved strength, pain, and QoL in BCS without increasing inflammation. After the follow-up period, inflammation-related protein levels decreased, and row strength gains were maintained, supporting the potential effects of concurrent training. Further larger and controlled studies are needed to confirm the results.

## Simple Summary

Breast cancer-related lymphedema (BCRL) is a common complication experienced by breast cancer survivors (BCS). The aim of this study was to analyze the effects of a 12-week supervised concurrent training program and a 12-week follow-up period without training on molecular, functional, and clinical outcomes in BCS with or at risk of BCRL. The 12-week concurrent training program improved muscle strength, pain perception, and quality of life-related outcomes in BCS without increasing inflammation. In addition, after the follow-up period, a significant decrease in various inflammation-related proteins was observed, and row strength gains were maintained.

## 1. Introduction

Breast cancer (BC) is the second most frequently diagnosed cancer worldwide. Approximately 2.3 million new cases occur each year [1], and the incidence is expected to rise to 3 million by 2040 [2]. Although the number of BC survivors (BCS) has increased, the experienced adverse health effects remain underexplored [3]. It is estimated that up to 90% of BCS experience long-term adverse effects including physical, functional, emotional, and psychosocial alterations that alter their quality of life (QoL). Pain, breast cancer-related lymphedema (BCRL) and reduced strength, cardiorespiratory fitness (CRF), and range of motion (ROM) represent the most common physical and functional changes experienced by BCS [4,5]. In fact, upper-body strength is generally lower in BCS compared with normative values [5]. Additionally, cardiotoxicity is a frequent complication in patients with BC undergoing chemotherapy and affects the QoL and mortality in BCS [6].

BCRL is a major and common complication in BCS that is characterized by the accumulation of extracellular fluid in the tissues due to damage to the lymphatic system [7]. Although axillary lymph node dissection (ALND) is considered to be the main risk factor for the development of BCRL, sentinel lymph node biopsy (SLNB) and radiotherapy are also recognized as contributing factors [8,9]. Indeed, the cumulative 5-year incidence rates for this complication are approximately 30.1% for ALND in combination with regional nodal radiotherapy, 24.9% for isolated ALND, 10.7% for SLNB with regional nodal radiotherapy, and 8.0% for SLNB alone [10]. A BMI ≥ 30 kg/m^2^ at the time of BC diagnosis, the presence of cellulitis, and a greater relative volume change following surgery are also well-established risk factors for BCRL [11]. This condition has a negative impact on QoL in BCS and is defined by increased limb volume, subcutaneous tissue thickness due to fat deposition, and inflammation [7]. The role of chronic inflammation in BCS is a major concern as it may contribute to the development and progression of the disease. Elevated C-reactive protein (CRP) and serum amyloid A levels have been associated with reduced survival in BCS. Therefore, the level of inflammation may represent an important marker of long-term survival in this population [12].

BC survivorship care may include strategies to reduce physical symptoms and improve cardiac health and overall well-being [13]. Among these, both aerobic and resistance exercise are proposed as safe and effective strategies to improve health-related outcomes in this population [14,15]. A recent systematic review demonstrated that combined training could increase muscle strength and CRF and improve several inflammatory markers [16]. This type of exercise is highly recommended in BC, possibly due to the benefits of combining aerobic and resistance training modalities [17,18]. Therefore, analyzing the effects of a concurrent training program (i.e., combining resistance and aerobic training within the same session) could provide valuable insight into its effects on clinical and functional parameters, with potential benefits for QoL in BCS. In addition, previous meta-analyses have shown that exercise can reduce the circulating levels of insulin-like growth factor 1 (IGF-1), interleukin (IL)-6, IL-10, tumor necrosis factor (TNF)-alpha (α), and CRP in patients with BC or BCS [19,20]. In fact, a significant linear interaction was observed between intervention length (>11 weeks) and changes in IL-6, suggesting a possible dose–response relationship [21]. A prior study demonstrated that a single resistance training session, regardless of the intensity used, did not increase the levels of proinflammatory markers in women with BCRL [22].

However, the long-term effects of exercise on inflammatory markers have not been previously confirmed in these patients. Additionally, most studies in patients with BCRL analyzed the effects of progressive and isolated resistance exercise [23,24] and did not include aerobic training. In fact, current exercise guidelines for lymphedema in cancer survivors only include specific recommendations for resistance exercise and not for aerobic or combined training [15].

Therefore, this single-arm study primarily aimed to investigate the impacts of concurrent training on upper- and lower-body strength and inflammation-related proteins in BCS with BCRL or at risk of developing it. In addition, the effects of the intervention on functional (handgrip strength and CRF) and clinical outcomes (body mass index [BMI], arm volume, subcutaneous and muscle thickness, ROM, physical activity levels, and heart rate variability [HRV]) were assessed along with self-reported measures of pain, function, and QoL after a 12-week concurrent training program and after a 12-week follow-up period without training.

## 2. Materials and Methods

### 2.1. Study Design

This single-arm quasi-experimental pilot study was conducted at the Miguel de Cervantes European University (Valladolid, Spain) between March 2024 and October 2024. The intervention comprised a 12-week supervised concurrent training program and a 12-week follow-up period without training. Molecular, functional, and clinical outcomes, including self-reported questionnaires, were assessed one week before (baseline) and after (post-intervention) the 12-week training program and one week after the 12-week follow-up period without training (follow-up). The study compiled with the standards of good clinical practice and the Declaration of Helsinki and was approved by the Ethics Committee for Drug Research of the Valladolid East Health Area on 23 November 2023 (PI 23-3382). All participants signed a written informed consent before the start of the study.

### 2.2. Study Participants

**Figure 1:**
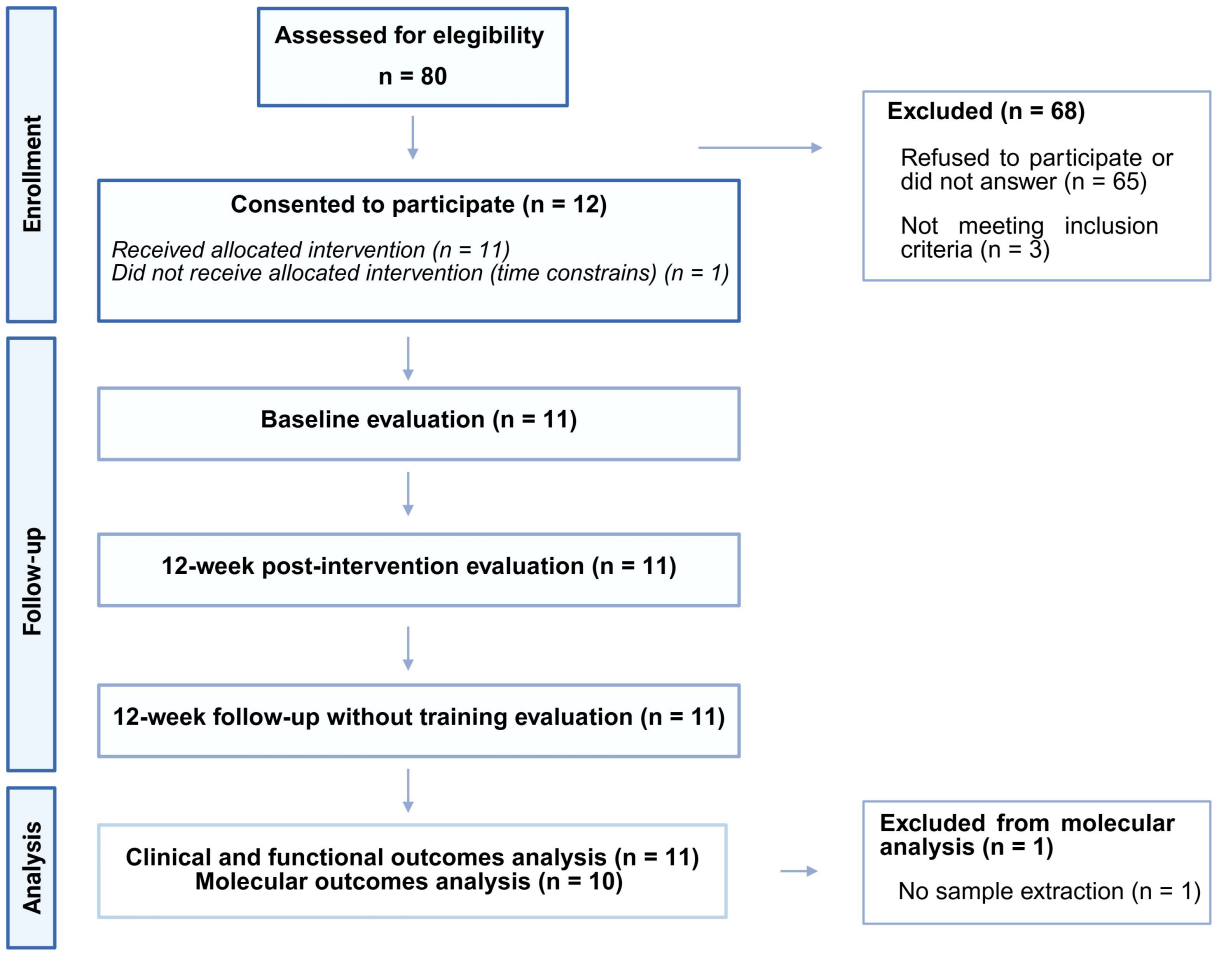
Flow of participants through the study.

A total of 80 BCS with an established clinical diagnosis of BCRL or at risk of developing it were initially invited to participate in the study from 20 December 2023 to 5 March 2024. Finally, 12 women agreed to participate and met the following inclusion criteria: (a) BCS aged 18–65 years old who had completed medical treatment (i.e., chemotherapy, radiotherapy, and/or surgery) at least six months prior to the study and (b) diagnosed with unilateral BCRL stage I or II as defined by the International Society of Lymphology (ISL) [25] or at risk of unilateral BCRL (who had undergone radical mastectomy/modified radical mastectomy/breast surgery and ALND/breast surgery and SLNB or breast surgery and axillary radiotherapy). The exclusion criteria were as follows: (a) recent surgery (less than three months) or planned surgery during the study duration; (b) cognitive impairment; and (c) health problems, pathologies, or diseases that prevented them from participating in the study. Finally, 11 BCS completed baseline, post-intervention, and follow-up assessments for functional, clinical, and self-reported outcomes. For the molecular analysis, one participant’s sample could not be collected at any time point and was not included in the statistical analysis of this outcome (Figure 1).

### 2.3. Exercise Training Program and Bandage Application

All participants completed a 12-week supervised and progressive concurrent training intervention and a 12-week follow-up period without training. After one week of comprehensive familiarization with the protocol in which individualized initial loading was proposed, the training program consisted of two face-to-face supervised exercise sessions per week of one hour each, conducted individually or in pairs based on participants’ availability, with a 1:1 supervision ratio in both cases. The session started with a 10 min warm-up consisting of mobility (scapulothoracic region, hip, knee, and ankle) and strength exercises (10 repetitions each of squats and front and lateral elevations with lightweight elastic band). Participants performed the resistance training using elastic resistance bands with seven different intensities (i.e., 5, 15, 20, 25, 30, 35, and 40 kg) and structured in a circuit-based format comprising seven functional exercises targeting all major muscle groups: (1) unilateral standing row, (2) unilateral standing chest press, (3) Pallof press, (4) squat, (5) single-leg deadlift, (6) unilateral front raise, and (7) step up. All exercises were tailored to the individual based on their height or widespan.

The participants completed two sets (seven exercises per set) of 8–12 repetitions of each exercise at a medium-to-high effort character [EC, determined as the relationship between the number of repetitions performed and the maximum achievable value [26]]. After completing the first circuit set, the participants rested for two min before beginning the next set. The rest between exercises was the minimum time required for the change of exercises. To monitor and adjust the intensity, the participants performed the first two sets of the first training session to muscle failure, which allowed an initial estimation of their individual strength capacity. The load was adjusted based on these sets to ensure an appropriate initial intensity. In addition, a re-evaluation of the training workload was conducted every three weeks using the Chronojump Force Sensor Kit (Chronojump Boscosystems^®^, Barcelona, Spain) and the open-source software Chronojump 2.3.0 for Windows (Chronojump Boscosystems^®^, Barcelona, Spain) to accurately quantify the mechanical load during each repetition. This ensured a progressive and individualized training stimulus. If they completed 17 to 20 repetitions, the exercise intensity was increased to the next resistance band level, and participants performed 10 repetitions with that proposed resistance. If they completed 16 repetitions or fewer, the number of repetitions was adjusted to 80% of that number of repetitions for the next training session without increasing the intensity.

After resistance training, participants performed aerobic exercise on a cycloergometer (Bodytone Acive Bike 200, Bodytone International Sport S.L., Murcia, Spain) at a moderate intensity [i.e., 70–75% of theoretical (calculated as 220 − age) maximum heart rate (HR)] [27]. HR was continuously monitored during each session using a chest strap Polar H10 (Polar Electro Oy, Kempele, Finland) and associated software (Polar Electro Oy, 2024; Kempele, Finland, version 3.5.8). At the beginning of the program, the duration of the aerobic exercise was set at 15 min. Every two weeks, the duration progressively increased by 5 min [27], up to a maximum of 30 min per session. Immediately at the end of each exercise session, the rate of perceived exertion (RPE) was assessed using a visual Borg CR-10 scale [28]. Participants decided whether to wear a compression garment during training or not, and they were encouraged to continue with their daily lifestyle during the length of the study. The details of the concurrent training intervention were also reported following the CORE-CERT guideline (Supplementary File S1, Supplementary Material), a tool designed to determine the core components of exercise interventions for BC [29].

Additionally, a cohesive compressive bandage was applied to participants with BCRL in the affected arm after each training session (Supplementary File S2, Supplementary Material). For reasons of comfort, this bandage was not applied to the participants at risk of developing BCRL. Participants were instructed to remove the bandage two hours after application [30]. An extensible cotton tubular bandage (Liderton^®^; sizes 5 and 6) was applied in contact with the skin. An elastic cotton bandage (NOBAFIX^®^; 4 cm × 4 m) was applied to the fingers and the dorsal area of the hand. The cohesive bandage (Cinfa Farmalastic^®^, 5 cm × 4.5 m for the hand and wrist and 10 cm × 4.5 m for the arm) was then applied, starting at the hand and extending to the proximal arm area. A cotton pad was placed at the antecubital fossa to improve comfort while wearing the bandage. The cohesive bandage was applied with an initial overlap of 50% at the most distal points and 25% from the elbow, ensuring that compression was highest in the distal region and gradually decreased toward the proximal segments [31].

### 2.4. Outcome Measurements

Primary outcomes included functional assessments (upper- and lower-body strength) and molecular analysis (inflammation-related proteins). Additionally, secondary clinical and functional outcomes and self-reported questionnaires were evaluated. All outcomes were measured at baseline, after the 12-week concurrent training program and after the 12-week follow-up period without. Adherence to the training sessions was reported as the percentage and number of sessions attended by the participants.

#### 2.4.1. Primary Outcomes

##### Functional Outcomes

• Upper- and lower-body strength

The maximal voluntary isometric contraction (MVIC) of both the upper and the lower body was measured using the Chronojump Force Sensor Kit (Chronojump Boscosystems^®^, Barcelona, Spain) and the open-source software Chronojump 2.3.0 for Windows (Chronojump Boscosystems^®^, Barcelona, Spain). The force signal was amplified to 80 Hz using a dedicated amplifier.

Upper-body MVIC was evaluated using the standing unilateral chest press and row exercises. For the unilateral standing chest press test, participants stood with both feet at the same height and at comfortable width, with the shoulder in 45° abduction and the elbow at 90° flexion, maintaining a neutral horizontal flexion. For the unilateral standing row, participants stood with both feet at the same height and comfortable width, the shoulder in a neutral position and the elbow in 90° flexion. For all upper body force measurements, the used handle was attached to a chain that was connected to the force sensor, which was attached to a height-adjustable bar. Both tests were performed first on the right arm, followed by the left.

Similarly, the lower-body MVIC was assessed using the isometric squat test. Participants were attached to the platform via a cable, which allowed for individual height adjustments. From the predetermined position (90° hip flexion and 90° knee flexion), participants were instructed to perform a maximal isometric contraction as fast as possible and hold it for five seconds. Researchers verbally encouraged the participants to ensure that each repetition was performed with maximal effort. For each exercise, three maximal attempts were performed for a minimum of five seconds with a 2 min rest period between measurements. Peak force was registered for each limb and attempt. The mean value of three trials was calculated for the statistical analysis.

##### Molecular Outcomes

• Inflammation-related proteins

Blood samples were collected by venipuncture of a peripheral forearm vein by an experienced nurse. Samples were collected in EDTA-treated Vacutainer tubes and centrifuged at 1500× *g* for 10 min. After the first centrifugation, the plasma was saved and centrifuged again at 2500× *g* for 15 min. Finally, 20 μL of plasma was transferred to cryogenic vials and stored at −80 °C. At the time of measurement, 20 μL of plasma of each sample was sent with dry ice to the Cobiomic laboratory of Olink ^®^ Proteomics, Córdoba, Spain, which analyzed 1 μL of each sample using the Olink ^®^ Target 96 Inflammation panel [Cobiomic Bioscience, S.L; https://olink.com/products/olink-target-96 (accessed on 7 June 2025)] and the proximity extension assay (PEA) to measure protein expression [32]. The PEA is based on pairs of antibodies linked to oligonucleotides with slight affinity for each other. The result of the assay is expressed as normalized protein expression (NPX), an arbitrary unit on a log_2_ scale. A higher value corresponds to a higher protein expression level. Each protein has a specific lower limit of detection (LOD), which is determined using negative controls included in each assay run [33]. Only proteins with NPX values above the LOD in at least 75% of the samples were included in the subsequent statistical analysis.

#### 2.4.2. Secondary Outcomes

##### Clinical Outcomes

• Body mass index

Height and weight were measured using a stadiometer and a Tanita BC-545N electronic scale (Tanita, Tokyo, Japan), wearing light clothing and without shoes. BMI was calculated (kg·m^−2^).

• Arm volume

Circumference measurements were taken on the affected and unaffected arms using a standard 1 cm tape measure (Orliman, Valencia, Spain) in direct contact with the skin and in the supine position with the shoulders abducted at 45° and the forearms supinated. Circumference measurements started at the metacarpophalangeal joints and were taken at 10 cm intervals (at 10, 20, 30, 40, and 50 cm) up the arm to the base of the axilla. Circumference measurements were collected and converted into volume using the truncated cone (*frustum*) formula [34]. The total arm volume of the affected and unaffected arm and the difference between the arms (in mL) were included in the subsequent statistical analysis.

• Tissue thickness

Subcutaneous and muscle thickness were measured on the affected arm using a Samsung HS30 ultrasound device (Samsung Healthcare Global, Gangwon, Republic of Korea) and a 40 mm, 3–12 MHz, linear-array transducer. The ultrasound measurement protocol is described in detail in Supplementary File S3, Supplementary Material. Briefly, muscle and subcutaneous tissue thickness was measured 10 cm distal and 10 cm proximal to the elbow. The transducer was placed perpendicular to the ventral axis of the upper limb, and sufficient gel was applied to avoid pressure on the arm during measurement [35]. Muscle thickness was defined as the distance from the highest point of the posterior fascia boundary to the highest point of the anterior fascia boundary portion. The thickness of the subcutaneous tissue was defined as the distance from the skin to the fascia. For all assessments, three images were acquired, and the mean value of both muscle and subcutaneous tissue was used for statistical analysis.

• Range of motion

Shoulder ROM for active flexion and abduction was measured using a conventional goniometer. ROM was assessed in both the affected and unaffected arm in a supine position, and three trials were recorded. The mean value was used for statistical analysis.

• Physical activity levels

Physical activity levels were objectively measured using the ActiGraph GT3X+ triaxial accelerometer (ActiGraph, Pensacola, FL, USA) [36]. At baseline, post-intervention, and follow-up assessments, all participants were instructed to wear the accelerometer attached to an elastic band over the right hip for seven days during all waking hours except for water-based activities. They also received verbal and written instructions and a wear time log to ensure adherence. The non-wear period was defined by the validated Choi algorithm [37]: 90 min window, 30 min stream frame, and 2 min spike tolerance. All data used for the analysis included at least 10 h of wear time for at least five days. Data were downloaded and analyzed in the ActiLife Software Version 6.13.4 (ActiGraph, Pensacola, FL, USA) using Freedson adult-specific cut points [38] in 60 s epochs: sedentary (<100 counts per min (cpm)), light physical activity (100–1951 cpm), and moderate-to-vigorous physical activity (MVPA) (≥1952 cpm). MVPA time was recorded for statistical analyses.

• Heart rate variability

HRV was measured using the Polar H10 HR sensor chest strap (Polar Electro Oy, Kempele, Finland) and the Elite HRV © app (Elite HRV Inc., Asheville, NC, USA). Each participant remained in a supine position for 15 min in a quiet and dark room with a controlled temperature (20–22 °C). The central 5 min of each measurement were recorded for statistical analysis. The Kubios HRV Scientific Little^®^ software (version 4.1.1., Kubios, Ltd., Kuopio, Finland) was used to remove the artefacts with a low threshold of beat corrections and to record the time domain measurements of HRV for analysis: mean R-R interval (Mean RR), standard deviation of normal-to-normal intervals (SDNN), and root mean square of successive differences (RMSSD). Moreover, the frequency measurements of low frequency (LF, 0.04–0.15 Hz) and high frequency (HF, 0.15–0.4 Hz) were also determined [39].

##### Functional Outcomes

• Cardiorespiratory fitness

A cardiopulmonary exercise test was performed with a cycloergometer (Wattbike AtomX, Wattbike, UK). To ensure patient safety, blood pressure was measured before and after the exercise test using the automated blood pressure measurement device Omron M7 (HEM-780, Omron, Kyoto, Japan), with the participant seated and in the starting position of the test.

The test protocol consisted of a 2 min rest period in the starting position, followed by a graded incremental exercise, starting at 50 W and increasing by 25 W every two minutes [40] until voluntary exhaustion or until 90% theoretical maximum HR ± 10% beats per min value was reached. After the main part, a 2 min cool-down was performed with the participants cycling without any resistance. HR and gas exchange were monitored during the whole protocol. Gas exchange was measured with a stationary spiroergometric device MetaLyzer 3B (Cortex Biophysik GmbH, Leipzig, Germany), which was calibrated each day of the exercise tests according to the manufacturer’s instructions. CRF (i.e., peak oxygen consumption [VO_2_peak]) was expressed relative to body weight (mL·min^−1^·kg^−1^).

• Handgrip strength

Handgrip strength was measured using the JAMAR^®^ Plus Smart Digital Hand Dynamometer (Patterson Medical Ltd., Sammons Preston, Nottinghamshire, UK). Participants sat on a chair, supported their forearms on a table, and held the dynamometer with the shoulder in a neutral position and the elbow in 90° degrees of flexion. They were asked to perform three maximal trials for three to five seconds with a 3 min rest period between attempts [41]. The handgrip test was performed first with the affected arm and subsequently with the unaffected arm. The mean value of the three trials was used for statistical analysis.

##### Self-Reported Questionnaires

• Pain

The intensity of the pain was assessed using a 0–10 numeric rating scale (NRS) [42]. Participants were asked to rate their pain in the affected arm at rest from 0, “no pain”, to 10, “worst pain imaginable”.

• Quality of life

The Functional Assessment of Cancer Therapy-Breast (FACT-B+4) was used to assess the health-related QoL [43]. This scale comprises 41 items corresponding to six domains (physical well-being, functional well-being, emotional well-being, social/family well-being, and BC- and arm-subscales). The total scores for FACT-B and FACT-B+4 were also calculated. Responses ranged from 0 (not at all) to 4 (very much), with higher scores reflecting lower symptom burden and a greater sense of overall well-being and QoL.

• Upper-body function

The 30-item Disabilities of the Arm, Shoulder and Hand (DASH) [44] questionnaire was administered to assess upper-body function. The DASH questionnaire assesses the physical function and symptoms of individuals with upper limb musculoskeletal disorders. Higher scores are associated with more functional limitations.

### 2.5. Statistical Analysis

All analyses were conducted using SPSS Statistics Version 23.0 (IBM Statistics, Chicago, IL, USA), Stata Statistical Software 14 (StataCorp, College Station, TX, USA), R version 4.4.2 (Pile of Leaves), and RStudio Desktop version 2024.12.0-467 for Windows (Posit Software, PBC, Boston, MA, USA). GraphPad Prism 8.0.1 was used to create plots. Baseline demographic and clinical data were summarized for all participants who completed the study baseline assessments. Continuous outcomes were reported as mean ± standard deviation (SD) and median and first and third quartiles, while categorical outcomes were expressed as percentages. Normality of continuous data was assessed using the Shapiro–Wilk test.

A one-way repeated-measures analysis of variance (ANOVA) was performed to evaluate the effects of time (baseline, post-intervention, and follow-up) on all primary and secondary outcomes with a normal distribution and the effects of time (weeks 1, 4, 7, and 10) on load progression data with a normal distribution. To reduce the risk of type I error, Bonferroni post-hoc analyses on primary and secondary outcomes were performed for pairwise comparisons when a significant time effect was observed. For outcomes with a non-normal distribution, the Friedman test was used to analyze the differences between the different time points. If a significant time effect was found, post-hoc pairwise comparisons were performed using the Wilcoxon signed-rank test. The effect size (ES) was reported as partial eta-squared (*η*^2^*p*) for ANOVAs and Kendall’s W for Friedman tests. The magnitude of changes between the different assessments was calculated using ES: (i) Cohen’s d (calculated as the mean change of baseline and post-intervention values divided by pooled SD) and 95% confidence interval (CI) for outcomes with normal distribution and (ii) the rank biserial correlation (r) and 95% CI for outcomes with a non-normal distribution (calculated with the formula r = Z/√N, where N is the total number of observations and the Z value of the Wilcoxon signed rank test).

Pearson’s correlations were performed to examine the associations between percentage changes (%Δ) in molecular, functional, and clinical outcomes and self-reported questionnaire scores that were significant at post-intervention compared with the baseline [%Δ calculated as ((post-intervention value − baseline value)/baseline value)*100] and those that were significant at follow-up compared with the baseline [%Δ calculated as ((follow-up value − baseline value)/baseline value)*100].

All results from statistical analyses were provided with the corresponding *p*-values. Statistical significance was defined as *p* < 0.05 for functional and clinical outcomes and self-reported questionnaires. A statistical significance of *p* < 0.001 was applied to the molecular data due to the high number of comparisons (74 proteins in total). However, considering the small sample size and the exploratory aim of this pilot study, no additional corrections for multiple comparisons were applied.

Fold change was determined by calculating the mean difference between the different time points (baseline, post-intervention, and follow-up) for each protein (NPX value, expressed in a log_2_ scale). Statistical significance (*p* < 0.001) was assessed using a paired *t*-test for proteins with a normal distribution and a Wilcoxon signed-rank test for proteins with a non-normal distribution.

Due to the pilot study design, a priori sample size calculation was not performed. Previous research suggests that a sample size of approximately 12 participants is recommended for pilot studies when no prior data are available to inform a power analysis [45]. In our study, 12 participants were initially recruited, and the final analyzed sample comprised 11 participants. To complement the interpretation of our findings, a post-hoc power analysis was performed using two approaches: (i) G*Power 3.1.9.7 (α = 0.05) in a single-group model with three measures converting *η*^2^*p* into Cohen’s f using the formula *f* = $\sqrt{\eta 2p/(1-\eta 2p)}$ [46] and (ii) Kendall’s W from the Friedman test and the noncentral chi-squared distribution using the formula χ^2^ = W × *n* × (k − 1) [47], where W is Kendall’s coefficient, *n* is the number of participants, and k is the number of measurements. Power was estimated in RStudio with α = 0.05.

## 3. Results

### 3.1. Participants’ Characteristics

All included participants were women and had a history of invasive ductal (81.82%) or lobular (18.18%) carcinoma diagnosis and had undergone breast and axillary surgery, with an average of 11 lymph nodes removed (Table 1).

Likewise, the majority had received prior radiotherapy (81.82%), chemotherapy (81.82%), and current or prior hormonal therapy (90.91%). A total of six participants had a clinical diagnosis of BCRL (stage I [18.18%] or II [36.36%]) and had completed the intensive phase of complex decongestive therapy (CDT). Four of them chose to wear their compression garments during the training sessions. No changes in BCRL self-care management were reported during the study. The average age was 53.00 ± 7.20 years, and the mean BMI at baseline was 25.42 ± 4.31 kg·m^−2^.

### 3.2. Adherence and Tolarance to Exercise Sessions

**Figure 2:**
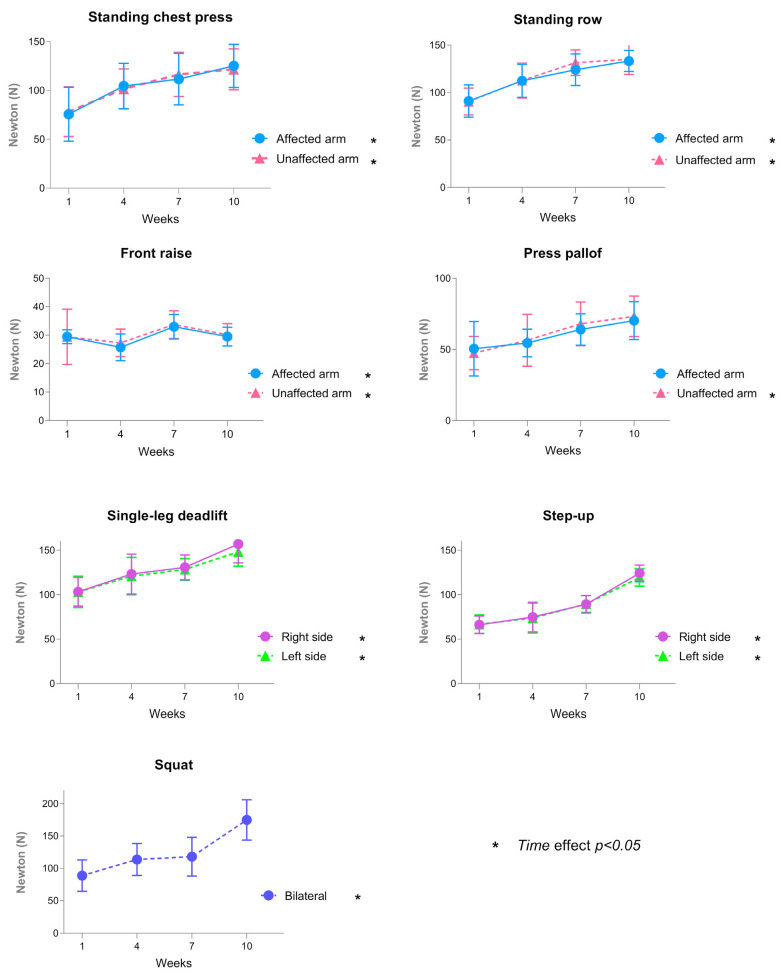
Progression in strength exercise load across the 12-week intervention, assessed using a force sensor at weeks 1, 4, 7, and 10. Exercises included standing chest press, standing row, front raise, Pallof press, single-leg deadlift, step-up, and squat.

Adherence to training sessions was 92.05%, with an average of 22 ± 3 of 24 possible sessions completed. No adverse events or BCRL onset or exacerbations were recorded during the study period. All sessions were accomplished with a moderate EC according to RPE (Borg CR-10 scale 6.26 ± 0.28 points) [26,48]. The average percentage of the theoretical maximum HR was 72.18 ± 1.01% during the aerobic part, which corresponded to a moderate intensity. During the intervention, the progression in resistance training intensity was systematically monitored and adjusted. As previously mentioned, a force sensor was used to objectively monitor changes in the training load every three weeks. The load was progressively increased by modifying the resistance band and/or adjusting the number of repetitions. Figure 2 represents the load progression through force sensor assessments (N) over the 12-week intervention. A significant time effect (*p* < 0.05) on the training intensity was shown for all exercises over the training weeks, except for the Pallof press on the affected arm.

### 3.3. Results of Primary Outcomes

#### 3.3.1. Functional Outcomes

**Figure 3:**
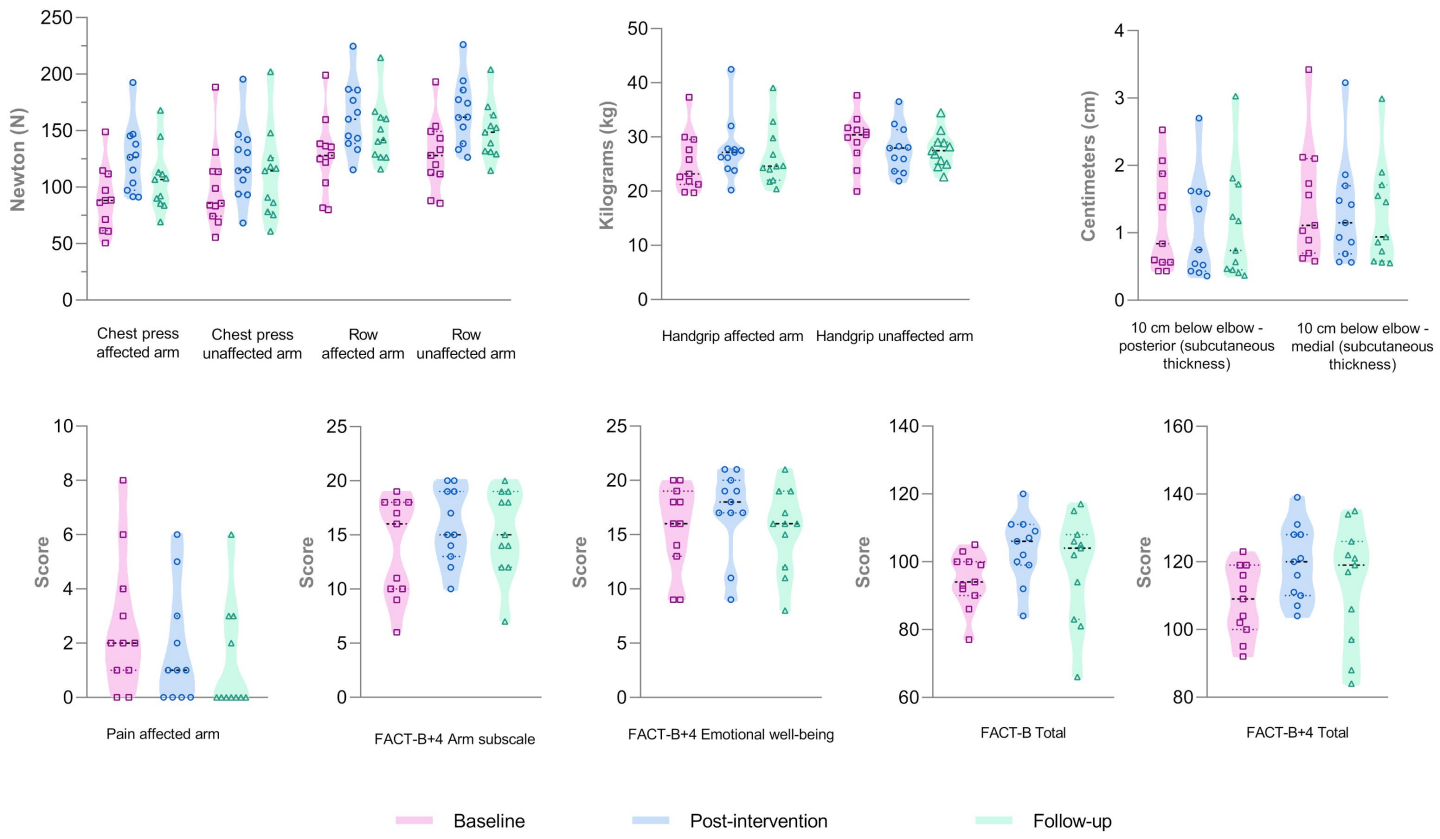
Functional and clinical outcomes with a significant time effect. Abbreviations. FACT-B+4, Functional Assessment of Cancer Therapy-Breast plus 4; cm, centimeters.

A significant time effect was observed in MVIC for unilateral chest press in both the affected (*p *< 0.001) and unaffected arm (*p *= 0.001) and for unilateral standing row also in the affected (*p *< 0.001) and unaffected arm (*p *< 0.001) (Figure 3; Supplementary File S4, Supplementary Material). In post-hoc analyses, a significant increase was observed in post-intervention compared with the baseline for unilateral chest press and unilateral row in both the affected (*p *< 0.001 and *p *= 0.004, respectively) and unaffected arm (*p *= 0.006 and *p *= 0.001, respectively). The post-hoc analyses also revealed that the increases in unilateral row remained significant at follow-up (*p *= 0.045 in the affected arm and *p *= 0.025 in the unaffected arm) compared with the baseline. In the squat test, a non-significant increase in MVIC was observed at the post-intervention assessment and at the follow-up.

#### 3.3.2. Molecular Outcomes

**Figure 4:**
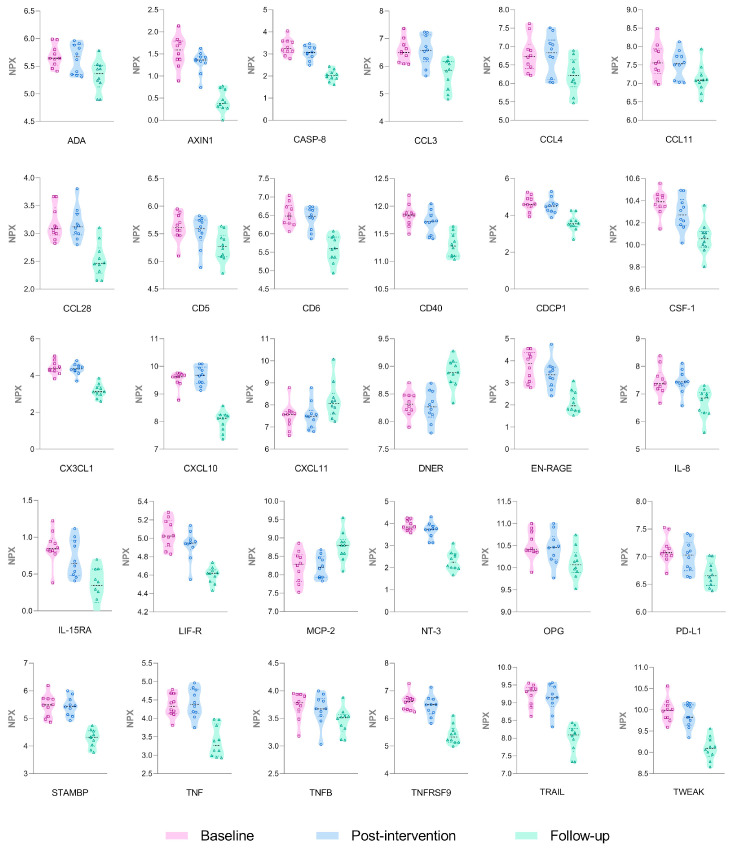
Proteins with a significant time effect.

A proteomic panel of 92 inflammation-related proteins was analyzed in plasma samples. However, 18 proteins had values below the LOD in ≥75% of samples and were not included in the statistical analysis. Samples from 10 participants were collected and analyzed at the baseline, post-intervention, and follow-up (Supplementary File S5, Supplementary Material). A total of 30 proteins showed a significant time effect (*p*<0.001) (Figure 4; Supplementary File S5, Supplementary Material).

Specifically, in post-hoc analyses, the following 19 proteins exhibited a significant decrease in the follow-up assessment compared with the baseline and post-intervention (*p *< 0.001): Axin-1 (AXIN1), caspase-8 (CASP-8), C-C motif chemokine 3 (CCL3), C-C motif chemokine 4 (CCL4), C-C motif chemokine 28 (CCL28), T-cell surface glycoprotein CD6 isoform (CD6), CD40L receptor (CD40), CUB domain-containing protein 1 (CDCP1), fractalkine (C3XCL1), protein S100-A12 (EN-RAGE), leukemia inhibitory factor receptor (LIF-R), neurotrophin-3 (NT-3), osteoprotegerin (OPG), programmed cell death 1 (PD-L1), STAM-binding protein (STAMBP), TNF, TNF receptor superfamily member 9 (TNFRSF9), TNF-related apoptosis-inducing ligand (TRAIL), and TNF (Ligand) superfamily member 12 (TWEAK). In addition, TNF-beta (TNFB), macrophage colony-stimulating factor-1 (CSF-1), and eotaxin (CCL11) levels were lower at follow-up compared with the baseline, and IL-8 and C-C motif chemokine 25 (CCL25) were reduced at follow-up compared with post-intervention (*p* < 0.001). In contrast, monocyte chemotactic protein 2 (MCP-2) and delta and notch-like epidermal growth factor-related receptor (DNER) showed a significant increase at follow-up compared with the baseline and post-intervention assessments (*p* < 0.001).

**Figure 5:**
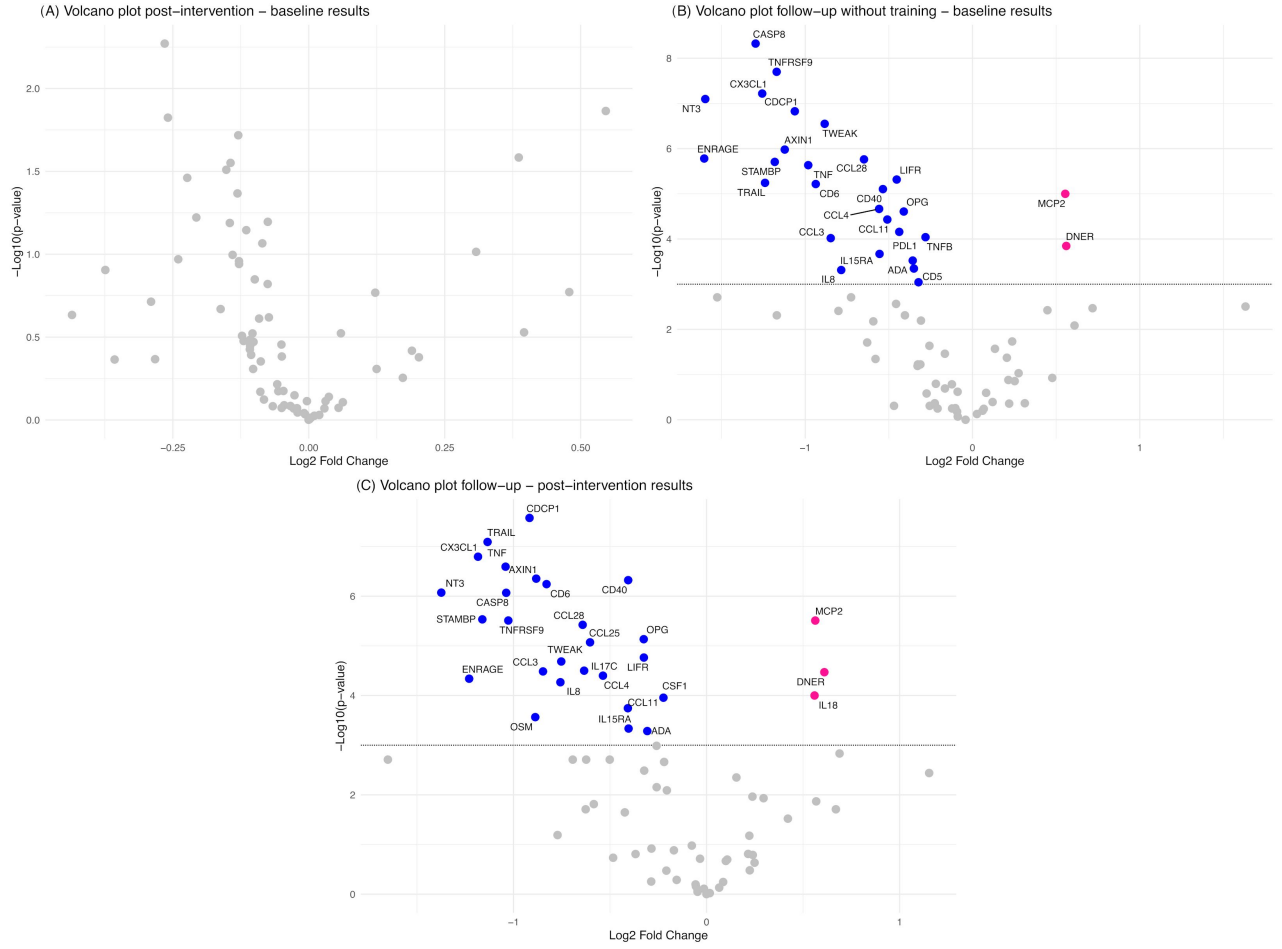
Volcano plots generated to compare protein levels (NPX) between (**A**) post-intervention versus baseline results, (**B**) follow-up versus baseline results, and (**C**) follow-up versus post-intervention results. The *y*-axis shows the log10 value of the *p*-values, and the *x*-axis shows the log2 fold change between the time points, with a negative fold change indicating a lower protein expression level in the post-intervention or follow-up assessments. The names of the proteins that showed a significant change (*p* < 0.001) are shown.

The mean value of 55 proteins decreased at post-intervention compared with the baseline, although no statistically significant differences were observed (Figure 5A). In addition to the above-mentioned proteins that showed a significant time effect and a significant change at *post hoc* analysis, a significant decrease was observed in adenosine deaminase (ADA), T-cell surface glycoprotein CD5 (CD5), IL-8, and IL-15 receptor subunit alpha (IL-15RA) at follow-up compared with the baseline (Figure 5B) (*p* < 0.001). Moreover, a significant decrease in ADA, IL-15RA, oncostatin M (OSM), and macrophage colony-stimulating factor 1 (CSF-1) was observed at follow-up compared with post-intervention (*p* < 0.001) (Figure 5C).

### 3.4. Results of Secondary Outcomes

#### 3.4.1. Clinical Outcomes

No significant changes were observed in BMI, arm volume, inter-arm volume difference, shoulder ROM, MVPA, or HRV-related outcomes (mean RR, SDNN, RMSSD, LF, and HF). By contrast, a significant time effect (*p *= 0.013) and reduction at follow-up assessment compared with the baseline was observed in medial subcutaneous tissue thickness distal to the elbow (*p *= 0.048). In addition, a significant time effect (*p *= 0.029) was observed in posterior subcutaneous tissue thickness (Figure 3; Supplementary File S4, Supplementary Material). However, the *post hoc* analysis did not reveal statistically significant results for this outcome.

#### 3.4.2. Functional Outcomes

The results for handgrip strength showed a significant time effect in the affected arm (*p *= 0.020), with a significant increase at post-intervention compared with the baseline (*p *= 0.021). Moreover, a significant time effect was observed in this outcome in the unaffected arm (*p* = 0.005), with a significant decrease at follow-up compared with the post-intervention assessment (*p *= 0.046) (Figure 3; Supplementary File S4, Supplementary Material). No significant changes were observed for VO_2peak_ (Supplementary File S4, Supplementary Material).

#### 3.4.3. Self-Reported Questionnaires

A significant time effect was observed for pain, measured through an NRS (*p *= 0.038). Specifically, pain decreased at post-intervention compared with the baseline (*p* = 0.005). In addition, a significant time effect was observed in the results of the FACT-B+4 questionnaire for arm subscale (*p *= 0.016), emotional well-being (*p *= 0.039), total FACT-B (*p *= 0.011), and FACT-B+4 (*p *= 0.003) scores. Except for arm subscale, the other results improved in *post hoc* analysis at post-intervention compared with the baseline (*p *= 0.027 for emotional well-being; *p* = 0.005 for total FACT-B; and *p* = 0.001 for total FACT-B+4 score). The results for the DASH questionnaire did not reveal a significant time effect (Supplementary File S4, Supplementary Material). ES estimations for all analyzed outcomes are reported in Supplementary Files S4–S6, Supplementary Material.

### 3.5. Correlation Between Outcomes

A positive and significant correlation was observed between the changes in chest press and row strength in both the affected (r = 0.915, *p* < 0.001) and unaffected arm (r = 0.656, *p* = 0.028) from baseline to post-intervention. The increase in FACT-B was also positively correlated with the increase in the FACT-B+4 (r = 0.937; *p* < 0.001) total score. As for the results that showed a significant change at follow-up compared with the baseline, the change in row strength of the affected arm was positively correlated with changes in CCL3 (r = 0.716; *p* = 0.020) and CCL11 (r = 0.779; *p* = 0.008). In addition, the changes in CCL3 (r = 0.687; *p =* 0.028), CSF-1 (r = 0.681; *p* = 0.030), MCP-2 (r = 0.658; *p* = 0.039), and DNER (r = −0.701; *p =* 0.024) were significantly correlated with changes in the subcutaneous tissue thickness at follow-up compared with the baseline (Supplementary File S7, Supplementary Material).

## 4. Discussion

**Figure 6:**
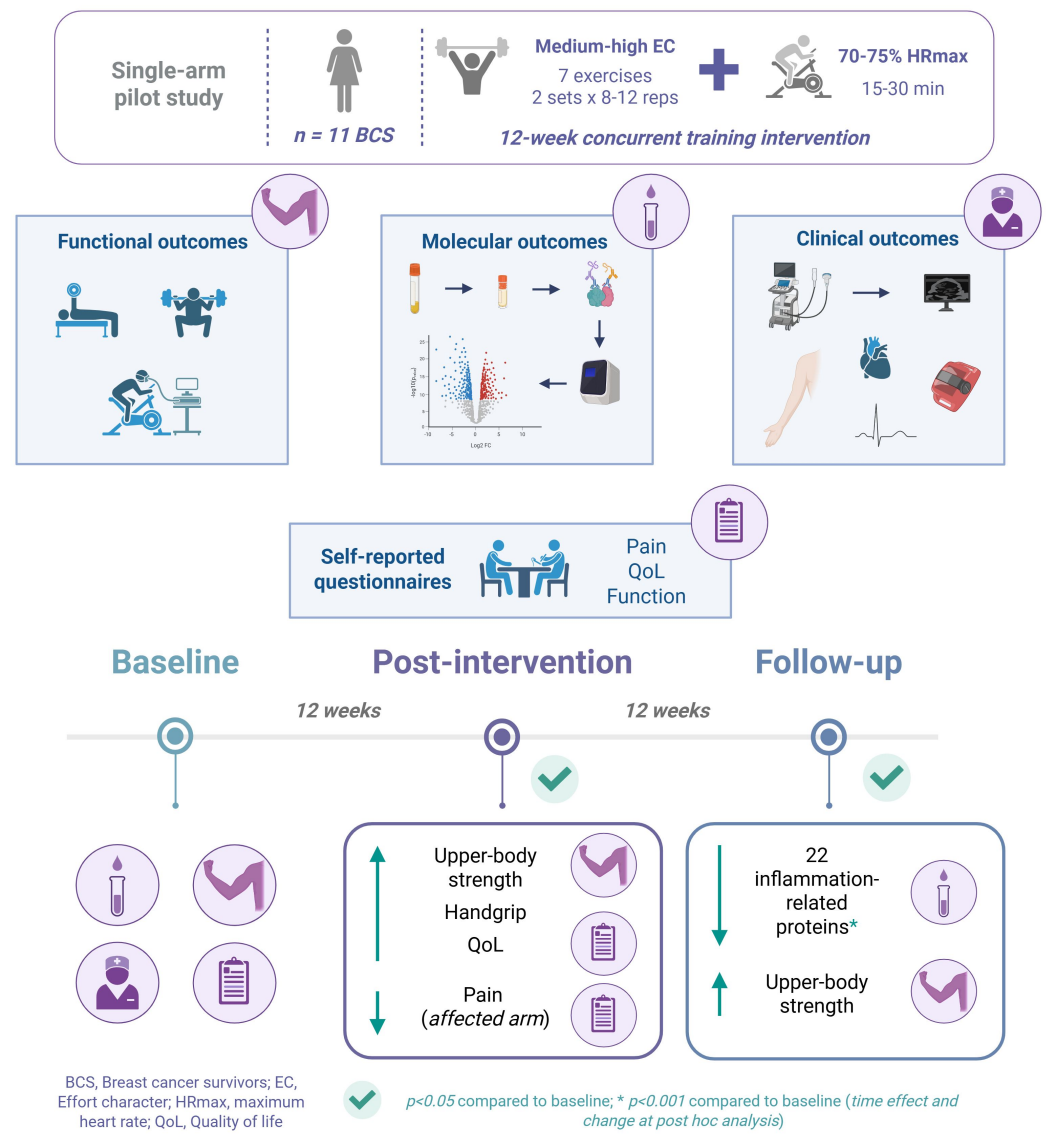
Main results obtained in the study.

The main findings of the present study were that 12 weeks of concurrent training in BCS with BCRL or at risk of developing it significantly improved upper-body strength (unilateral chest-press and unilateral row MVIC) of both arms, handgrip strength of the affected arm, pain, emotional well-being, and total QoL. At post-intervention, no significant changes were observed in the inflammation-related proteins included in the analysis. However, a decreasing trend was observed in most of them (the mean value of 55 of the 74 analyzed proteins decreased at post-intervention compared with the baseline). In addition, after a 12-week follow-up period without training, the increase in row MVIC was maintained, and a significant time effect and decrease at *post hoc* analysis was observed in various inflammation-related proteins (AXIN1, CASP-8, CCL3, CCL4, CCL11, CCL28, CD6, CD40, CDCP1, CSF-1, C3XCL1, EN-RAGE, LIF-R, NT-3, OPG, PD-L1, STAMBP, TNF, TNFB, TNFRSF9, TRAIL, and TWEAK) compared with the baseline (Figure 6). No adverse events, BCRL onset, or exacerbations were observed throughout the study.

Systemic inflammation is an important long-term prognostic factor for BCS as elevated inflammation levels are associated with an increased risk of comorbidities and cancer recurrence [49]. Additionally, large-scale transcriptional profiling studies in patients with cancer-associated lymphedema have shown that both RNA and circulating protein expression profiles have a distinct inflammatory signature, supporting the role of chronic inflammation in the disease [7]. A previous study conducted in 21 BCS with BCRL showed that acute resistance exercise performed at different intensities did not increase inflammation or markers of muscle damage [50]. However, no study to date has focused on analyzing the chronic effects of exercise on proinflammatory markers in BCS with BCRL or at risk of developing it. Our study provides new insights into the effects of 12 weeks of concurrent training and 12 weeks without training on this outcome. In particular, the expression levels of 22 inflammation-related proteins showed a significant time effect and decreased significantly in post-hoc analysis at the follow-up assessment compared with the baseline: AXIN1, CASP-8, CCL3, CCL4, CCL11, CCL28, CD6, CD40, CDCP1, CSF-1, C3XCL1, EN-RAGE, LIF-R, NT-3, OPG, PD-L1, STAMBP, TNF, TNFB, TNFRSF9, TRAIL, and TWEAK.

Six of these 22 proteins belong to the TNF and TNF receptor superfamily: OPG, TNF, TNFB, TNFRSF9, TRAIL, and TWEAK. Evidence has suggested that these proteins are active in inflammatory and autoimmune disease by activating the nuclear factor kappa B (NF-κB) signaling [51], which suggests a role in patients with BCRL or at risk [52]. Other proteins belong to the CC (CCL3, CCL4, CCL11, and CCL28) and CX3C (C3XCL1) chemokine family [53]. Specifically, CCL3, CCL4, CCL11, and CCL28 are considered proinflammatory chemokines [53,54]. Any disruption in cytokine and chemokine signaling can contribute to inflammatory diseases and cancer as chemokines selectively recruit and maintain proinflammatory cells throughout the chronic inflammatory response [55,56].

Furthermore, PD-L1 is upregulated on different types of cancer, including BC, in response to proinflammatory cytokines [57]. CD6, CD40, and CDCP1 are also involved in the pathogenesis of impaired immune states [58], increased inflammation through the increase in reactive oxygen species production and chemokine expression [59], and poor BC prognosis [60]. Moreover, CSF-1 is highly expressed in immune cells and plays a central role in the progress of various inflammatory diseases [61]. AXIN1 promotes inflammation by activating the Stress-Activated Protein Kinase/Jun N-terminal Kinase pathway, which triggers the release of pro-inflammatory genes [62]. In addition, CASP-8 is involved in a variety of inflammatory diseases, including immune system disorders and cancer [63]. EN-RAGE is considered an important circulating proinflammatory marker as ligation of this protein with the receptor for advanced glycation end products activates several signaling processes such as nuclear factor-κB (NF-κB) and mitogen-activated protein kinase, which increases the production of proinflammatory cytokines [64]. LIFR is a complex receptor as many ligands have the potential to interact and activate different signaling pathways. Binding of leukemia inhibitory factor to LIFR activates the protein kinase-B (Akt)-mammalian target of rapamycin (mTOR) signaling pathway, which can promote tumorigenesis and metastasis [65]. In addition, NT-3 activates the NT-3 growth factor receptor, a tropomyosin receptor kinase that is overexpressed in BC and activates the phosphatidylinositol 3-kinase (PI3K)-Akt pathway, which promotes cell proliferation [66]. This PI3K-Akt pathway is also involved in inflammation and has been associated with lymphatic dysfunction in patients with primary lymphedema [67]. STAMBP is correlated with poor prognosis in patients with triple-negative BC [68].

The decrease in these inflammation-related proteins suggests that concurrent training may induce a protective effect that is evident during the follow-up period, which may be due to delayed physiological adaptations. A recent meta-analysis demonstrated that crucial growth-related and inflammatory markers, including TNF-α, IGF-1, IL-6, CRP, and IL-10, were decreased by exercise. However, the results varied according to the type of exercise and the duration of the training period (less than or equal to 12 weeks) [20]. Exercise can reduce inflammatory indicators by enhancing endothelial function and insulin sensitivity. Conversely, acute responses to exercise may lead to short-term variations in inflammation levels, whereas sustained exercise results in longer-term inflammation reduction [49]. This might explain why the benefits observed in our study occurred after the 12-week follow-up period and not immediately at the post-intervention assessment. However, compared with the baseline, exercise did not substantially increase inflammation at the end of the intervention, highlighting the safety of concurrent training in BCS with or at risk of BCRL. A prior exercise study found that TNF-α plasma levels were lower in BCS after six months of follow-up compared with the baseline, which might support exercise’s long-term anti-inflammatory benefits [69]. However, future research should include larger sample sizes to corroborate the observed effects in BCS with BCRL or at risk as several studies have demonstrated an immediate anti-inflammatory response after an exercise program of combined training in BCS and patients with BC [70,71]. Although a significant interaction has been reported between the intervention length (>11 weeks) and changes in IL-6 [21], a recent review proposed that exercise interventions lasting longer than 16 weeks may induce greater effects in BCS [18], suggesting a possible dose–response relationship. However, the optimal exercise length to achieve sustained benefits in BCS remains unknown. Future research should include longer-term interventions to determine the sustainability of exercise-induced changes in inflammation-related outcomes among BCS.

Previous research has demonstrated that elevated circulating levels of proinflammatory cytokines are associated with reduced muscle mass and strength in adults. Specifically, IL-6 plays a dual role in muscle metabolism by promoting either anabolism or catabolism depending on the context. However, prolonged exposure to IL-6 and the concomitant activation of other cytokines may contribute to muscle atrophy by impairing anabolic processes and disrupting energy homeostasis, as well as directly mediating muscle catabolism [72]. In fact, it is hypothesized that elevated levels of circulating proinflammatory cytokines may contribute to muscle wasting in cancer survivors [73]. Patients with BC may exhibit lower strength scores compared with healthy women both before and after treatment [74], which may be associated with increased joint dysfunction. Consistent with previous research focused on analyzing the effects of resistance exercise [23], our findings support the hypothesis that implementing a concurrent training program—combining resistance and aerobic exercise—significantly enhances upper-body strength in BCS with BCRL or at risk of developing it. This increase may also protect the arm from injuries by minimizing mechanical stress during daily life activities [75].

Regarding lower-body strength, although the increase was not statistically significant, a mean difference of 232.38 N was observed between baseline and post-intervention assessments, which could be clinically relevant for this population. A recent randomized controlled trial in patients with BC undergoing adjuvant therapy found that improvements in one-repetition maximum leg press after a 12-week resistance training program were associated with increased walking capacity and exhaustion time during incremental walking, which has implications for functional performance [76]. This increase in lower-body strength, measured by leg press, was also confirmed in a previous meta-analysis conducted in patients with BCRL or at risk [75]. The lack of significant improvements in lower-body strength might be related to the use of the isometric squat test, which differs from the leg press or knee extension typically used in previous studies [23,75]. Handgrip strength is also a distinct component of upper-body strength [77] and is considered a relevant health marker [78]. Indeed, in BCS, reduced handgrip strength correlates with decreased upper-limb functionality [79]. Our results showed a significant increase in handgrip strength of the affected arm after 12 weeks of concurrent training, which could therefore have clinical implications.

Self-reported pain decreased significantly at post-intervention compared with the baseline. Although pain perception represents a subjective outcome that may be associated with potential response bias, the NRS is considered appropriate for the assessment of this outcome as it has adequate sensitivity to change and is useful in the research setting [80]. In addition, the total QoL score, assessed by the FACT-B+4 questionnaire, improved significantly after the concurrent training intervention, which aligns with the results of a previous randomized controlled 12-week resistance training trial in patients with BC undergoing adjuvant radiotherapy [81]. A meta-analysis also showed that patients with BCRL and high ACSM compliance experienced a higher improvement in QoL compared with patients with low compliance [82], which highlights the importance of including specific exercise interventions in this population. Although subjective, QoL is influenced by BCRL diagnosis and progression [83], emphasizing the need to consider patient-reported outcomes when evaluating the impacts of interventions.

Regarding tissue thickness and arm volume, no significant changes were observed at post-intervention, although a significant reduction was observed in subcutaneous tissue thickness in the lateral region of the forearm at follow-up assessment compared with the baseline. Moreover, it is important to note that the thickness of the subcutaneous tissue decreased at post-intervention in all regions assessed, which could be clinically relevant. The lack of significant changes at post-intervention could be due to heterogeneity in the accumulation of lymphatic fluid and fibrosis in different areas and regions of the arm in each participant. A previous study showed that 8 weeks of resistance training significantly reduced subcutaneous tissue thickness in patients with BCRL [35], emphasizing the potential effect of exercise on this outcome. Further research is needed to analyze the specific changes in tissue thickness in studies including exercise interventions, in which muscle growth should be considered [23] and isolated assessment of arm volume may not be sufficient. Although circumference measurements are useful to monitor changes in BCRL severity, the formulas developed to calculate volume may overestimate arm volume compared with other methods such as water displacement [34]. The ISL recommends additional clinical assessment by imaging modalities, including ultrasound to detect changes in tissue composition and techniques to detect changes in lymphatic drainage [25]. In the present study, all participants with BCRL had already completed the intensive phase of CDT, which could represent greater stability in BCRL severity in terms of arm volume. In fact, maintenance phase interventions aim to preserve volume reduction and prevent recurrence, rather than to achieve additional therapeutic effects [84].

Results from the present study indicate that concurrent training was well tolerated and improved functional, molecular, and self-reported related outcomes in BCS with BCRL or at risk of developing it. In addition, the intervention did not induce BCRL exacerbations in terms of arm volume, tissue thickness, or inflammation. However, baseline status including BMI [85] and diet may have influenced the molecular analysis. Indeed, participants’ dietary intake was not monitored during the study period, which may have influenced the results, as previous research suggests that various aspects of diet are associated with plasma proteins [86]. Another limitation of the present study is the small sample size of participants. Although the power analysis results indicated that the sample (*n* = 11) was sufficient to detect large effects, the small number of participants may have limited the ability to detect smaller changes in other outcomes. In addition, the single-arm study design is suitable for analyzing the feasibility of the proposed training program, but the effect of this intervention should be interpreted with caution due to the lack of a control group. However, the aim of this study was to provide preliminary evidence through a novel and long-term analysis of inflammation-related proteins. A prospective randomized controlled trial with a larger sample size would confirm the observed results and their interpretation since the presence of a control group with usual care would allow the detection of interactions between the groups.

The results of the present study provide new and preliminary insights into the benefits of concurrent training in BCS with or at risk of BCRL. The functional results align partially with previous research showing that resistance training significantly improves both upper- and lower-body strength in this population [23,75]. In our study, significant improvements were observed only in upper-body strength. In addition, the molecular findings were consistent with previous research that demonstrated that resistance training did not increase the levels of inflammation-related markers 24 h after exercise sessions of different intensities [22]. A recent review also showed that the combination of resistance and resistance exercises offers benefits for the prevention and management of BCRL, especially when programs are adapted to individual needs [87].

## 5. Conclusions

A 12-week supervised concurrent training program significantly improved the upper-body and handgrip strength, pain, physical and emotional well-being, and QoL in BCS with or at risk of developing BCRL. At post-intervention, a non-significant decreasing trend was observed for most of the analyzed inflammation-related proteins. In addition, after a 12-week follow-up period without training, molecular changes were observed and improvements in muscle strength were maintained, emphasizing the potential role of this type of training in improving BCS recovery. The training program was well tolerated and did not lead to adverse events. However, the single-arm study design and the small analyzed sample size limit the ability to evaluate the true effectiveness of the intervention, and further randomized controlled trials are needed to investigate and confirm the observed results.

## Figures and Tables

**Table 1:** Baseline characteristics of participants.

		Breast Cancer Survivors (*n* = 11)
Age—years (mean ± SD)		53.00 ± 7.17
Weight—kg (mean ± SD)		65.65 ± 12.59
Height—m (mean ± SD)		1.61 ± 0.06
BMI—kg·m^−2^ (mean ± SD)		25.42 ± 4.31
Time since cancer diagnosis—years (mean ± SD)		6.83 ± 4.56
Affected arm	Right—*n* (%)	5 (45.45)
	Left—*n* (%)	6 (54.55)
Breast surgery—*n* (%)		11 (100)
Tumorectomy—*n* (%)		5 (45.45)
Tumorectomy + simple mastectomy—*n* (%)		1 (9.09)
Radical mastectomy—*n* (%)		3 (27.27)
Modified radical mastectomy—*n* (%)		2 (18.18)
Axillary surgery—*n* (%)		11 (100)
SLNB—*n* (%)		2 (18.18)
ALND—*n* (%)		8 (72.73)
SLNB + ALND—*n* (%)		1 (9.09)

Abbreviations: ALND, axillary lymph node dissection; BMI, body mass index; SD, standard deviation; SLNB, sentinel lymph node biopsy.

## Data Availability

Data sets generated and/or analyzed during the present study are not publicly available due to data protection but can be requested from the corresponding author upon reasonable request.

## Supplementary Materials

The following supporting information can be downloaded at https://www.mdpi.com/article/10.3390/cancers17121967/s1: Supplementary File S1. CORE-CERT checklist; Supplementary File S2. Cohesive bandage application; Supplementary File S3. Protocol for assessing muscle and subcutaneous tissue thickness using ultrasound; Supplementary File S4. Baseline, post-intervention, and follow-up values in functional and clinical outcomes and self-reported questionnaires (*n* = 11 breast cancer survivors); Supplementary File S5. Baseline, post-intervention, and follow-up protein level (NPX) values (*n* = 10 breast cancer survivors); Supplementary File S6. Estimated effect size [Cohen’s d and 95% confidence interval and the rank biserial correlation (r) and 95% confidence interval]; Supplementary File S7. Correlations between outcomes.

## Abbreviations

The following abbreviations are used in this manuscript:

ADA	Adenosine deaminase
Akt	Protein kinase-B
ALND	Axillary lymph node dissection
ANOVA	Analysis of variance
AXIN1	Axin-1
BC	Breast cancer
BCRL	Breast cancer-related lymphedema
BMI	Body mass index
CASP-8	Caspase-8
CCL3	C-C motif chemokine 3
CCL4	C-C motif chemokine 4
CCL11	Eotaxin
CCL25	C-C motif chemokine 25
CCL28	C-C motif chemokine 28
CDT	Complex decongestive therapy
CD5	T-cell surface glycoprotein CD5
CD6	T-cell surface glycoprotein CD6 isoform
CD40	CD40L receptor
CDCP1	CUB domain-containing protein 1
C3XCL1	Fractalkine
CRF	Cardiorespiratory fitness
CRP	C-reactive protein
CSF-1	Macrophage colony-stimulating factor 1
DASH	Disabilities of the arm, shoulder, and hand
DNER	Delta and notch-like epidermal growth factor-related receptor
EC	Effort character
EN-RAGE	Protein S100-A12
FACT-B+4	Functional Assessment of Cancer Therapy-Breast
HF	High frequency
HR	Heart rate
HRV	Heart rate variability
IGF-1	Insulin-like growth factor 1
IL	Interleukin
ISL	International Society of Lymphology
IL-15RA	IL-15 receptor subunit alpha
LF	Low frequency
LIF-R	Leukemia inhibitory factor receptor
LOD	Limit of detection
MCP-2	Monocyte chemotactic protein 2
mTOR	Mammalian target of rapamycin
MVIC	Maximal voluntary isometric contraction
MVPA	Moderate-to-vigorous physical activity
NF-κB	Nuclear factor-κB
NRS	Numeric rating scale
NT-3	Neurotrophin-3
OPG	Osteoprotegerin
OSM	Oncostatin M
PD-L1	Programmed cell death 1 ligand 1
PEA	Proximity extension assay
PI3K	Phosphatidylinositol 3-kinase
QoL	Quality of life
RMSSD	Root mean square of successive differences
ROM	Range of motion
RPE	R rate of perceived exertion rate of perceived exertion
SD	Standard deviation
SDNN	Standard deviation of normal-to-normal intervals
SLNB	Sentinel lymph node biopsy
STAMBP	STAM-binding protein
TNF	Tumor necrosis factor
TNFB	TNF-beta
TNFRSF9	TNF receptor superfamily member 9
VO2peak	Peak oxygen consumption
