# Supplementary material for: The Effects of Concurrent Training on Molecular, Functional, and Clinical Outcomes in Breast Cancer Survivors: A Pilot Study
Source: Cancers (Basel). 2025 Jun 13;17(12):1967. doi: 10.3390/cancers17121967 (PMC12191275; doi:10.3390/cancers17121967)
Supplement: Supplementary file 1 [file cancers-17-01967-s001.zip › cancers-3669226-supplementary.pdf]

**The effects of concurrent training on molecular, functional and clinical outcomes in breast cancer survivors: A pilot study**

Celia García-Chico[1], Susana López-Ortiz[1*], Salvador Santiago-Pescador[2], Paloma Guillén-Rogel[2], Saúl Peñín-Grandes[1], Lisa Musso-Daury[1], Francisco Javier Iruzubieta-Barragán[2], José Pinto-Fraga[1], Sergio Maroto-Izquierdo[1,3], Lourdes del Río Solá[4], Alejandro Santos-Lozano[1,5]

1 i+HeALTH Strategic Research Group, Department of Health Sciences, Miguel de Cervantes European University, 47012 Valladolid, Spain
2 Department of Health Sciences, Miguel de Cervantes European University, 47012 Valladolid, Spain
3 Proporción A, Applied Sports Science Center, 47015 Valladolid, Spain
4 Vascular Surgery Department, University Hospital of Valladolid, 47003 Valladolid, Spain
5 Physical Activity and Health Research Group ("PaHerg"), Research Institute of Hospital "12 de Octubre" ("imas12"), 28041 Madrid, Spain
* Correspondence: slopezo@uemc.es; Tel.: +34 983001000

**SUPPLEMENTARY MATERIAL**

*Legends*

**Supplementary file S1.** CORE-CERT Checklist.

**Supplementary file S2.** Cohesive bandage application.

**Supplementary file S3.** Protocol for assessing muscle and subcutaneous tissue thickness using ultrasound.

**Supplementary file S4**. Baseline, post-intervention and follow-up values in functional and clinical outcomes and self-reported questionnaires (n = 11 breast cancer survivors).

**Supplementary file S5.** Baseline, post-intervention and follow-up protein expression levels (NPX) values (n = 10 breast cancer survivors).

**Supplementary file S6**. Estimated effect size [Cohen's d and 95% confidence interval and the rank biserial correlation (r) and 95% confidence interval].

**Supplementary file S7.** Correlations between outcomes. Table S7.1. Pearson correlation analysis between changes (%Δ) in outcomes that showed a significant time effect and a significant change at post-intervention compared to baseline *(based on* post-hoc *analysis)*: Affected and unaffected arm-related outcomes and quality of life (n = 11 breast cancer survivors). Table S7.2. Pearson correlation analysis between changes (%Δ) in outcomes that showed a significant time effect and a significant change at post-intervention compared to baseline *(based on* post-hoc *analysis)*: Quality of life-related outcomes (n = 11 breast cancer survivors). Table

S7.3. Pearson correlation analysis between changes (%Δ) in outcomes that showed a significant time effect and a significant change at follow-up compared to baseline *(based on* post-hoc *analysis)*: Molecular, clinical and functional outcomes (n = 10 breast cancer survivors).

**Supplementary file S1**. CORE-CERT Checklist[1] 4

**Supplementary file S2.** Cohesive bandage application. 8

Figure S2.1. Extensible cotton tubular bandage. 8

Figure S2.2. Elastic cotton bandage. 8

Figure S2.3. Cohesive bandage for hand and wrist. 9

Figure S2.4. Cohesive bandage for the arm. 9

**Supplementary file S3.** Protocol for assessing muscle and subcutaneous tissue thickness using ultrasound. 10

Figure S3.1. Subcutaneous tissue thickness 10 cm distal to the elbow: Anterior. 10

Figure S3.2. Subcutaneous tissue and muscle thickness 10cm distal to the elbow: Medial. 11

Figure S3.3. Subcutaneous tissue thickness 10cm distal to the elbow: Posterior. 11

Figure S3.4. Subcutaneous tissue and muscle thickness 10cm distal to the elbow: Lateral. 12

Figure S3.5. Subcutaneous tissue and muscle thickness 10cm proximal to the elbow: Anterior. 12

Figure S3.6. Subcutaneous tissue thickness 10cm proximal to the elbow: Medial. 13

Figure S3.7. Subcutaneous tissue thickness 10cm proximal to the elbow: Lateral. 13

Figure S3.8. Subcutaneous tissue and muscle thickness 10cm proximal to the elbow: Posterior. 14

**Supplementary file S4**. Baseline, post-intervention and follow-up values in functional and clinical outcomes and self-reported questionnaires (n = 11 breast cancer survivors) 15

**Supplementary file S5**. Baseline, post-intervention and follow-up protein expression levels (NPX) values (n = 10 breast cancer survivors). 22

**Supplementary file S6.** Estimated effect size [Cohen's d and 95% confidence interval and the rank biserial correlation (r) and 95% confidence interval]. 31

**Supplementary file S7.** Correlations between outcomes 38

**Table S7.1.** Pearson correlation analysis between changes (%Δ) in outcomes that showed a significant time effect and a significant change at post-intervention compared to baseline (based on post-hoc analysis): Affected and unaffected arm-related outcomes and quality of life (n = 11 breast cancer survivors) 38

**Table S7.2.** Pearson correlation analysis between changes (%Δ) in outcomes that showed a significant time effect and a significant change at post-intervention compared to baseline (based on post-hoc analysis): Quality of life-related outcomes (n = 11 breast cancer survivors) 39

**Table S7.3.** Pearson correlation analysis between changes (%Δ) in outcomes that showed a significant time effect and a significant change at follow-up compared to baseline (based on post-hoc analysis): Molecular and functional outcomes (n = 10 breast cancer survivors) 40

**Supplementary file S1**. CORE-CERT Checklist[1]

| Section/Topic | Item # | Checklist item | Location |
|---|---|---|---|
| **WHAT - Materials** | **1** | Detailed description of the type of exercise equipment (e.g. free weights, dumbbells, resistance bands, exercise equipment such as machines, treadmill, bicycle ergometer, etc) preferably with information on brand names or producer when identifiable. | 2.3. Exercise training program and bandage application. *"Participants performed the strength training using elastic resistance bands with seven different intensities […]. Participants performed aerobic exercise on a cycloergometer (Bodytone Acive Bike 200, Bodytone International Sport S.L., Murcia, Spain) at a moderate intensity"* |
| **HOW – Individually or group** | **2a** | Describe whether exercises are performed individually or in a group, including information on group size. | 2.3. Exercise training program and bandage application. *"The training program consisted of two face-to-face supervised exercise sessions per week of one hour each, conducted individually or in pairs based on participants' availability, with a 1:1 supervision ratio in both cases"* |
| **HOW – Supervision** | **2b** | Describe whether exercises are supervised or unsupervised. | |
| **HOW – Mode of delivery** | **2c** | Describe the modes of delivery (e.g., face to face or by some other mechanism, such as Internet or telephone) of the intervention. | |
| **HOW – Decision rule for determining progression** | **3a** | Detailed description of the decision rule(s), which defines when or at which state exercise progressions were made or started | 2.3. Exercise training program and bandage application. *"In addition, a re-evaluation of the training workload was conducted every three weeks […] to accurately quantify the mechanical load during each repetition. […] At the beginning of the program, the duration of the aerobic exercise was set at 15 min. Every two weeks, the duration progressively* |
| **HOW – Progression** | **3b** | Detailed description of how the exercise program was progressed or modified (e.g., for of an increase or decrease in volume, intensity, frequency, rest intervals etc., or stimulus modifications, moving from simple to more advanced exercises) using specific information or data | |

| | | | |
|---|---|---|---|
| | | | *increased by 5 min (24), up to a maximum of 30 min per session."* |
| **HOW – Replication of each exercise** | **4a** | Detailed description of each exercise to enable replication, including information on targeted muscles or common exercise names and equipment used | |
| **HOW – Home program exercise-components** | **4b** | Detailed description of any additional home program exercise-components being not an integral part of the exercise intervention (e.g., other exercises, stretching, functional tasks, etc.) with information fulfilling item 13 for each exercise, if not<br>applicable, then it should be stated that subjects were encouraged not to make any lifestyle changes during the study or similar instructions | 2.3. Exercise training program and bandage application.<br>*"Participants decided whether to wear a compression garment during training or not, and they were encouraged to continue with their daily lifestyle during the length of the study."* |
| **HOW – Nonexercise components (information material)** | **5a** | Describe whether there are any exercise or training informational materials, manuals, or written instructions used | NA |
| **HOW – Nonexercise components (interventions)** | **5b** | Describe whether there are any nonexercise components or strategies as part of the intervention (e.g., education, cognitive behavioural therapy, massage etc.), if not applicable, then it should be stated that subjects were encouraged not to make any lifestyle changes during the study or similar instructions | 2.3. Exercise training program and bandage application.<br>*"Additionally, a cohesive compressive bandage was applied to participants with BCRL in the affected arm after each training session."* |
| **HOW MUCH – Frequency** | **6a** | Detailed description of the frequency of each exercise | 2.3. Exercise training program and bandage application.<br>*"The training program consisted of two face-to-face supervised exercise sessions per week of one hour each"* |
| **HOW MUCH – Volume** | **6b** | Detailed description of the intensity of each exercise (e.g., load, repetitions, sets, rest intervals between reps or sets, speed, HR, $VO_2max$, BORG scale etc.) | 2.3. Exercise training program and bandage application. |
| **HOW MUCH – Time (intervention duration)** | **6c** | Detailed description of the duration of the exercise program | 2.3. Exercise training program and bandage application.<br>*"The training program consisted of two face-to-face supervised exercise sessions* |

| | | | |
|---|---|---|---|
| | | | *per week of one hour each. […]After strength training, participants performed aerobic exercise on a cycloergometer […]. At the beginning of the program, the duration of the aerobic exercise was set at 15 min. Every two weeks, the duration progressively increased by 5 min (24), up to a maximum of 30 min per session. Immediately at the end of each exercise session, the rate of perceived exertion (RPE) was assessed using a visual Borg CR-10 scale (26).”* |
| **HOW MUCH – Time (overall session duration)** | **6d** | Detailed description of the duration of the session duration (including warm-up and cooldown if information available) | 2.3. Exercise training program and bandage application.<br>*“The training program consisted of two face-to-face supervised exercise sessions per week of one hour each […].*The session started with a 10-min warm-up consisting of mobility”. |
| **HOW MUCH – Time (duration of different modalities)** | **6e** | Detailed description of the duration of different modalities, if applicable (e.g., combined aerobic and resistance training) | 2.3. Exercise training program and bandage application.<br>The detailed description is given during the whole section. |
| **HOW MUCH – Type/Training** | **6f** | Detailed description of the training modalities | |
| **HOW MUCH – Procedures** | **6g** | Detailed description of the training sequence or schedule of sessions, including warm-up and cooldown | |
| **TAILORING – Generic or individual** | **7a** | Describe whether the exercises are generic (1 size fits all) or tailored | 2.3. Exercise training program and bandage application.<br>The training load was adjusted based on baseline assessments and the exercises were tailored to the |
| **TAILORING – How tailored to individual** | **7b** | Detailed description of how exercises are tailored to the individual | |

| | | | |
|---|---|---|---|
| | | | individual based on the height or widespan. |

[1]*Bünzen C, Knuth J, Bucher M, Weisser B, Schmidt T. CORE-CERT Items as a Minimal Requirement for Replicability of Exercise Interventions: Results From Application to Exercise Studies for Breast Cancer Patients. J Strength Cond Res. 2023;37(5):e346-e360.*

**Supplementary file S2.** Cohesive bandage application.

1. **Extensible cotton tubular bandage (Liderton®; sizes 5 and 6).**

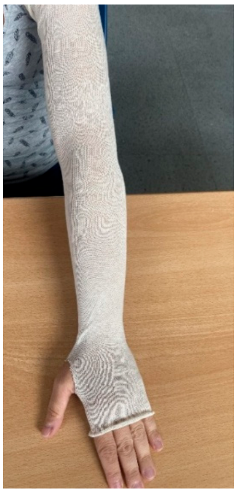

Figure S2.1. Extensible cotton tubular bandage.

2. **Elastic cotton bandage (NOBAFIX®; 4cm x 4m)**

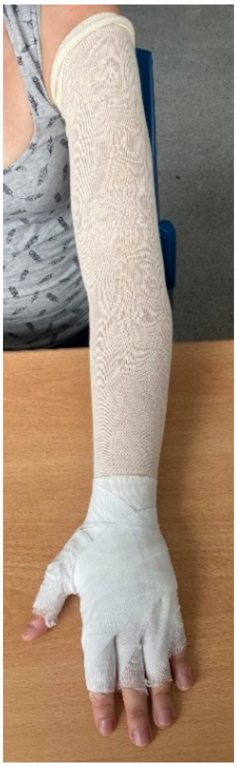

Figure S2.2. Elastic cotton bandage.

3. **Cohesive bandage (Cinfa Farmalastic®; 5 cm x 4.5 m for the hand and wrist)**

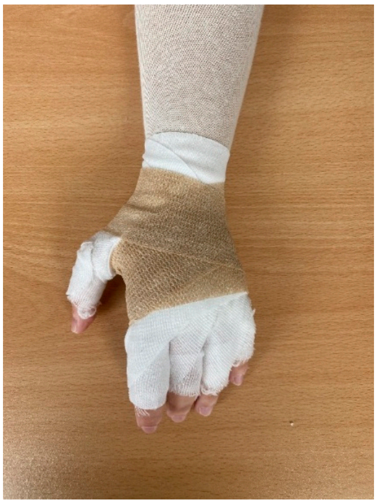

Figure S2.3. Cohesive bandage for hand and wrist.

4. **Cohesive bandage (Cinfa Farmalastic®; 10 cm x 4.5 m for the arm)**

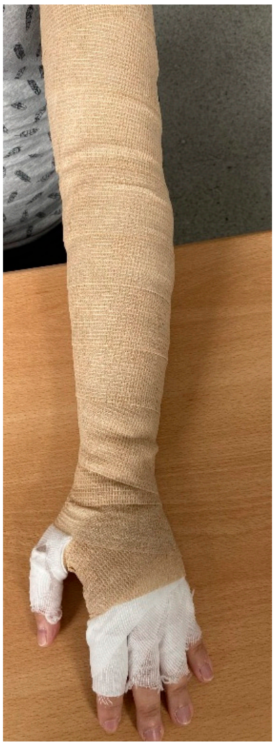

Figure S2.4. Cohesive bandage for the arm.

**Supplementary file S3.** Protocol for assessing muscle and subcutaneous tissue thickness using ultrasound.

**Participant's position**: Supine and lateral decubitus position.

**Protocol**: The assessor marked the anterior (a), medial (b), posterior (c), and lateral (d) areas of the participants' arm at 10 cm distal and proximal to the elbow to measure the tissue thickness. The transducer was placed perpendicular to the ventral axis of the upper limb with minimal pressure. Muscle thickness was defined as the distance from the highest point of the posterior fascia to the highest point of the anterior fascia boundary portion. The thickness of the subcutaneous tissue was defined as the distance from the skin to the fascia. For all assessments, three images were acquired and the mean value of both muscle and subcutaneous tissue was calculated for subsequent statistical analysis.

**Described protocol**:

**Measurement 10cm distal:**

- *10 cm distal anterior*: Supine decubitus position. With the elbow in extension, forearm in supination and the hand resting on a table, the mid-point of the distance (in cm) between the epicondyle and the epitrochlea was marked. A measurement was made between the ulnar and radial styloids (anterior) and the midpoint was marked. Both midpoints were connected using a tape measure and a measurement point was marked 10 cm distal from the flexure of the elbow (midpoint between the epicondyle and the epitrochlea).

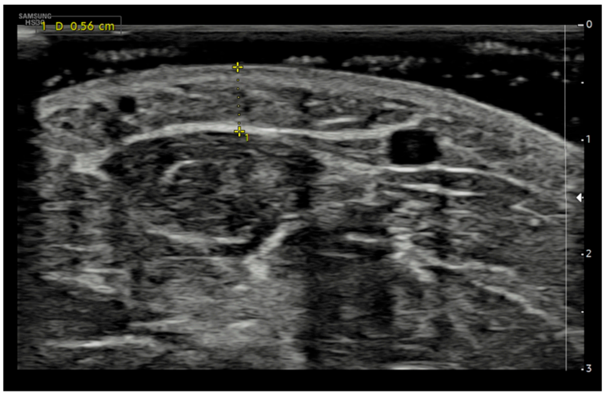


Figure S3.1. Subcutaneous tissue thickness 10 cm distal to the elbow: Anterior.

- *10 cm distal medial:* Supine decubitus position. With the elbow extended, forearm in supination, and the hand resting on a table, the epitrochlea was connected to the ulnar styloid, and a measurement point was marked 10 cm distal from the epitrochlea.

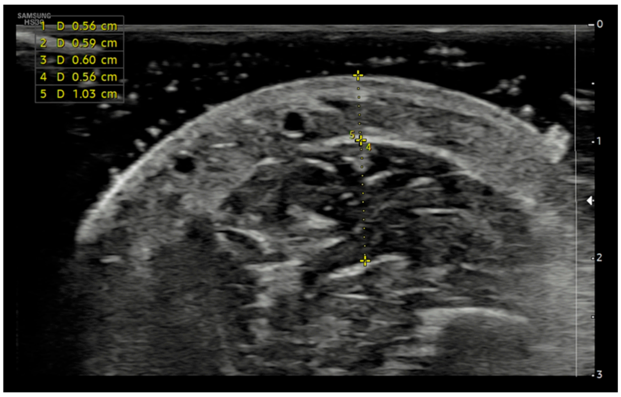


Figure S3.2. Subcutaneous tissue and muscle thickness 10cm distal to the elbow: Medial.

- *10 cm distal posterior*: Supine decubitus position. With the elbow in flexion and the hand resting on the shoulder, the most cranial point of the olecranon was marked. A measurement was made between the ulnar and radial (posterior) styloids, and the midpoint was marked. Both points (olecranon and styloid midpoint) were connected using a tape measure, and a measurement point was marked 10 cm distal to the olecranon.

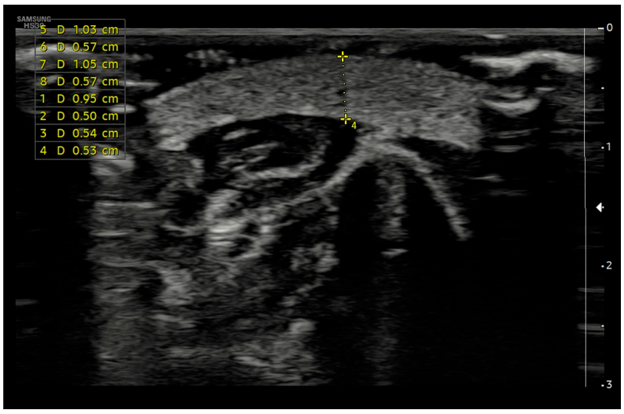


Figure S3.3. Subcutaneous tissue thickness 10cm distal to the elbow: Posterior.

- *10 cm distal lateral*: Supine decubitus position. With the elbow extended and the hand resting on a table, the epicondyle was connected to the radial styloid, and a measurement point was marked 10 cm distal from the epicondyle.

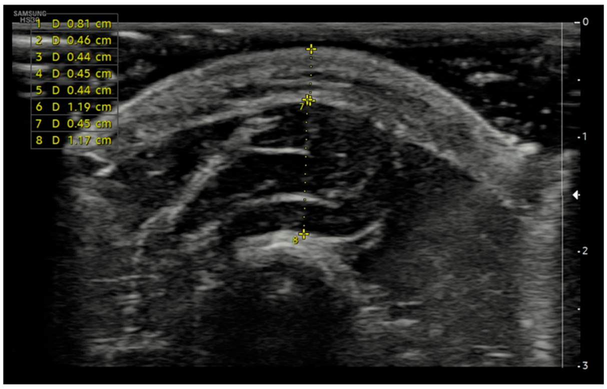


Figure S3.4. Subcutaneous tissue and muscle thickness 10cm distal to the elbow: Lateral.

**<u>Measurement 10cm proximal:</u>**

- *10 cm proximal anterior*: Supine decubitus position. With the elbow in extension and the hand resting on a table, forearm in supination, a measurement was made in cm of the distance between the epicondyle and the epitrochlea, and the midpoint was marked. A point was also marked at the level of the acromion. Both midpoints were connected using a tape measure, and a measurement point was marked 10 cm proximal to the midpoint between the epicondyle and the epitrochlea.

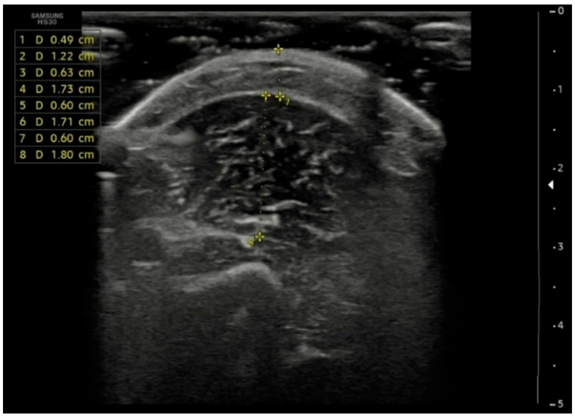


Figure S3.5. Subcutaneous tissue and muscle thickness 10cm proximal to the elbow: Anterior.

- *10 cm proximal medial:* Supine decubitus position. With the elbow in extension, the epitrochlea was connected to the acromioclavicular joint (ACC), and a measurement point was marked 10 cm proximal to the epitrochlea.

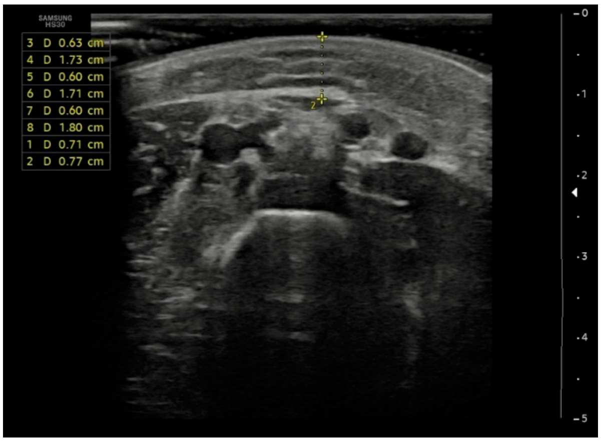


Figure S3.6. Subcutaneous tissue thickness 10cm proximal to the elbow: Medial.

- *10 cm proximal lateral*: Lateral decubitus position. With the elbow in extension and the forearm in supination, the epicondyle was attached to the acromion, and a measurement point was marked 10 cm proximal to the epicondyle.

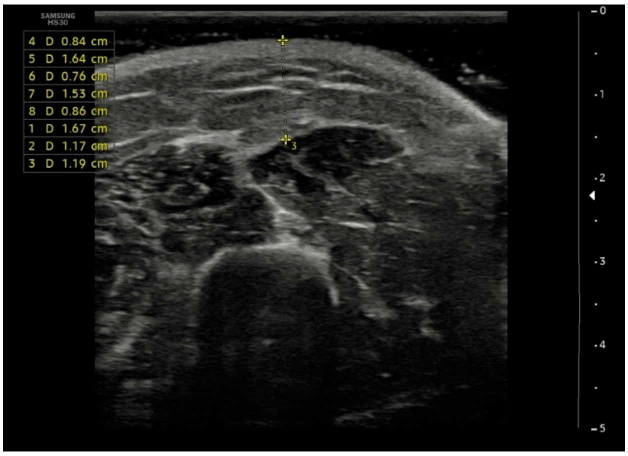


Figure S3.7. Subcutaneous tissue thickness 10cm proximal to the elbow: Lateral.

- *10 posterior proximal posterior*: Lateral decubitus position. With the elbow in extension, the distance between the marks "ACC joint – epitrochlea" and "acromion – epicondyle" was measured at the dorsal region of the arm. In the direction of this longitudinal line, the points were connected with a tape measure, and a measurement point was marked 10 cm proximal to the most cranial and prominent point of the olecranon.

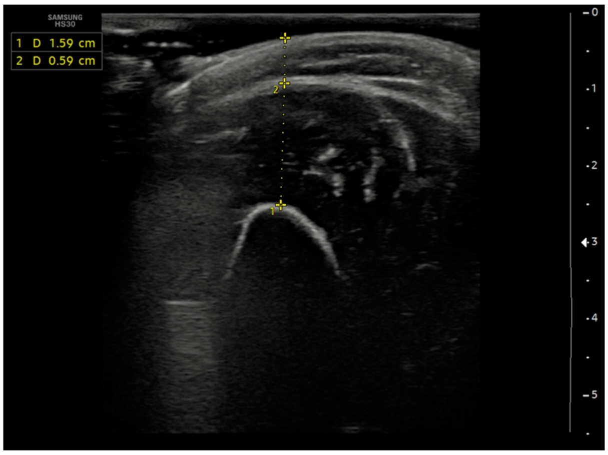


Figure S3.8. Subcutaneous tissue and muscle thickness 10cm proximal to the elbow: Posterior.

**Supplementary file S4**. Baseline, post-intervention and follow-up values in functional and clinical outcomes and self-reported questionnaires (n = 11 breast cancer survivors).

| **Outcome** | **Baseline** Mean±SD / **Baseline** Median [Q1 - Q3] | **Post-intervention** Mean±SD (% change from baseline) / **Post-intervention** Median [Q1 - Q3] | **Follow-up** Mean±SD (% change from baseline) / **Follow-up** Median [Q1 - Q3] | **Time** *p-value* | **Effect size** (*partial eta-squared or Kendall's W*) | ***Post-hoc* statistical Power** |
|---|---|---|---|---|---|---|
| **Unilateral chest press (affected arm; N)** | 89.09 ± 28.61 | 125.09 ± 30.39** (+48.05%) | 106.65 ± 25.52+ (+25.66%) | ***<0.001*** | 0.678 | +99% |
| | 88.33 [66.45 - 104.53] | 126.42 [100.43 - 141.60] | 106.84 [87.82 - 112.32] | | | |
| **Unilateral chest press (unaffected arm; N)** | 99.78 ± 36.77 | 121.77 ± 33.85* (+26.91%) | 110.68 ± 39.80 (+13.22%) | ***0.001*** | 0.498 | +99% |
| | 85.70 [78.76 - 113.81] | 115.27 [100.11 - 137.57] | 114.44 [82.31 - 122.10] | | | |
| **Unilateral row (affected arm; N)** | 128.13 ± 33.69 | 161.45 ± 30.85* (+30.59%) | 148.70 ± 27.54*+ (+20.23 %) | ***<0.001*** | 0.584 | +99% |
| | 128.07 [112.87 - 137.35] | 160.06 [140.96 - 181.37] | 141.73 [127.78 - 161.17] | | | |
| **Unilateral row (unaffected arm; N)** | 129.03 ± 30.92 | 166.62 ± 29.31** (+32.70%) | 149.16 ± 24.59*+ (+18.72%) | ***<0.001*** | 0.651 | +99% |
| | 127.92 [112.36 - 146.30] | 162.05 [145.90 - 181.68] | 148.62 [131.69 - 159.07] | | | |
| **Bilateral squat (N)** | 747.06 ± 257.51 | 979.44 ± 289.22 (+41.81%) | 856.11 ± 242.50 (+23.15%) | *0.066* | 0.272 | 98.89% |

| | | | | | | |
|---|---|---|---|---|---|---|
| | 688.78 [554.28 - 939.11] | 949.29 [779.60 - 1062.71] | 774.55 [685.22 - 1012.10] | | | |
| **Handgrip strength (affected arm; kg)**[a] | 25.33 ± 5.40 | 27.75 ± 5.70* (+10.45%) | 26.39 ± 5.54 (+4.61%) | ***0.020*** | 0.355 | 70.65% |
| | 23.20 [21.52 - 28.58] | 27.13 [25.17 - 27.73] | 24.60 [23.02 - 28.28] | | | |
| **Handgrip strength (unaffected arm; kg)** | 27.78 ± 4.34 | 29.54 ± 4.71 (+6.47%) | 27.63 ± 3.32+ (0.00%) | ***0.005*** | 0.409 | +99% |
| | 27.93 [24.80 - 29.82] | 30.40 [28.03 - 31.50] | 27.47 [25.30 - 29.00] | | | |
| **MVPA (minutes/week)**[a] | 353.64 ± 165.48 | 302.64 ± 127.27 (-12.37%) | 314.36 ± 171.13 (-7.88%) | *0.336* | 0.099 | 24.26% |
| | 298.00 [237.50 - 390.00] | 288.00 [200.0 - 379.50] | 279.00 [208.50 – 400.00] | | | |
| **$VO_{2peak}$ (ml·min-1·kg-1)** | 25.27 ± 4.63 | 25.64 ± 4.08 (+2.39%) | 24.82 ± 4.81 (-1.72%) | *0.483* | 0.070 | 43.92% |
| | 26.00 [22.50 - 27.50] | 27.00 [23.00 - 29.00] | 26.00 [22.50 - 28.00] | | | |
| **Arm volume (affected arm; ml)**[a] | 2271.92 ± 661.57 | 2296.14 ± 715.94 (+0.67%) | 2257.84 ± 694.57 (-0.99%) | *0.078* | 0.231 | 51.06% |
| | 2071.14 [1890.60 - 2324.67] | 2097.80 [1891.45 - 2349.71] | 2050.19 [1894.02 -2293.84] | | | |
| **Arm volume (unaffected arm; ml)** | 2034.87 ± 417.97 | 2055.63 ± 422.14 (+1.10%) | 2047.21 ± 449.75 (+0.51%) | *0.643* | 0.030 | 20.30% |
| | 1996.60 [1715.08 - 2317.15] | 1980.57 [1778.58 - 2275.38] | 1999.86 [1736.23 - 2324.99] | | | |
| **Inter-arm volume difference (ml)** | -237.04 ± 416.03 | -240.51 ± 462.85 (-126.54%) | -210.62 ± 464.31 (-6.09%) | *0.354* | 0.099 | 60.27% |

| | | | | | | |
|---|---|---|---|---|---|---|
| | -118.10 [-239.04 - -13.95] | -76.97 [-213.78 - -22.93] | -15.78 [-189.82 - 33.10] | | | |
| **Shoulder flexion (affected arm; degrees)[a]** | 167.97 ± 10.33 | 169.00 ± 7.22 (+0.80%) | 168.82 ± 13.08 (+0.60%) | *0.933* | 0.006 | 6.00% |
| | 167.33 [160.50 - 178.00] | 170.67 [163.00 - 172.00] | 173.33 [165.00 - 178.67] | | | |
| **Shoulder flexion (unaffected arm; degrees)[a]** | 171.18 ± 10.12 | 174.36 ± 4.20 (+2.14%) | 173.94 ± 4.13 (+1.85%) | *0.846* | 0.015 | 7.58% |
| | 177.33 [163.50 - 178.33] | 174.00 [172.33 - 177.33] | 174.00 [170.67 - 177.33] | | | |
| **Shoulder abduction (affected arm; degrees)[a]** | 162.42 ± 21.88 | 171.33 ± 7.88 (+7.17%) | 168.18 ± 13.52 (+4.58%) | *0.803* | 0.020 | 8.46% |
| | 175.33 [145.83 - 179.83] | 171.33 [168.67 - 176.33] | 175.00 [163.33 - 178.83] | | | |
| **Shoulder abduction (unaffected arm; degrees)[a]** | 174.42 ± 9.29 | 176.06 ± 4.49 (+1.16%) | 171.85 ± 10.96 (-1.43%) | *0.607* | 0.045 | 13.18% |
| | 179.67 [172.50 - 180.00] | 176.67 [174.50 - 179.50] | 178.00 [164.00 - 180.00] | | | |
| **BMI (kg·m$^{-2}$)** | 25.42 ± 4.31 | 25.40 ± 4.26 (0.00%) | 25.35 ± 4.16 (-0.13%) | *0.952* | 0.005 | 6.98 % |
| | 24.11 [22.94 - 29.28] | 24.24 [22.94 - 29.25] | 24.31 [23.18 - 28.82] | | | |
| **ULTRASOUND AFFECTED ARM** | | | | | | |
| **10 cm below elbow – anterior (subcutaneous thickness -cm)** | 1.14 ± 0.47 | 1.08 ± 0.46 (-4.96%) | 1.08 ± 0.50 (-6.24%) | *0.168* | 0.163 | 85.26 % |
| | 0.94 [0.88 - 1.38] | 0.89 [0.72 - 1.35] | 0.95 [0.80 - 1.31] | | | |
| | 1.44 ± 0.86 | 1.31 ± 0.78 | 1.26 ± 0.76* | ***0.013*** | 0.354 | +99% |

| | | | | | | |
|---|---|---|---|---|---|---|
| | | (-7.08%) | (-12.37%) | | | |
| **10 cm below elbow – medial (subcutaneous thickness -cm)** | 1.11 [0.80 - 1.34] | 1.15 [0.78 - 1.28] | 0.94 [0.66 - 1.2] | | | |
| **10 cm below elbow – medial (muscle thickness -cm)** | 1.02 ± 0.37 | 1.02 ± 0.38 (+1.42%) | 0.91 ± 0.28 (-7.11%) | *0.098* | 0.207 | 94.03% |
| | 1.00 [0.78 - 1.59] | 0.90 [0.79 - 1.01] | 0.83 [0.78 - 0.92] | | | |
| **10 cm below elbow – posterior (subcutaneous thickness -cm)[a]** | 1.17 ± 0.75 | 1.08 ± 0.75 (-9.15%) | 1.09 ± 0.83 (-8.60%) | ***0.029*** | 0.322 | 66.10% |
| | 0.84 [0.56 - 1.72] | 0.75 [0.48 - 1.60] | 0.74 [0.46 - 1.48] | | | |
| **10 cm below elbow – lateral (subcutaneous thickness -cm)[a]** | 1.03 ± 0.70 | 0.91 ± 0.50 (-6.84%) | 0.93 ± 0.49 (-4.33%) | *0.599* | 0.053 | 14.80% |
| | 0.83 [0.64 - 1.16] | 0.83 [0.61 - 1.00] | 0.94 [0.55 - 1.08] | | | |
| **10 cm below elbow – lateral (muscle thickness -cm)** | 1.13 ± 0.33 | 1.23 ± 0.24 (+18.51%) | 1.16 ± 0.24 (+15.38%) | *0.389* | 0.080 | 49.84% |
| | 1.16 [1.11 - 1.27] | 1.30 [1.10 - 1.37] | 1.19 [1.08 - 1.32] | | | |
| **10 cm above elbow – anterior (subcutaneous thickness -cm)[a]** | 1.06 ± 0.39 | 1.00 ± 0.43 (-6.25%) | 1.07 ± 0.43 (+1.01%) | *0.105* | 0.205 | 46.10% |
| | 0.92 [0.85 - 1.14] | 0.81 [0.75 - 1.11] | 1.00 [0.77 - 1.21] | | | |
| **10 cm above elbow – anterior (muscle thickness -cm)** | 1.49 ± 0.30 | 1.61 ± 0.28 (+8.30%) | 1.58 ± 0.36 (+6.16%) | *0.104* | 0.203 | 93.01% |
| | 1.44 [1.36 - 1.70] | 1.59 [1.44 - 1.79] | 1.64 [1.43 - 1.76] | | | |
| | 1.77 ± 0.93 | 1.62 ± 0.83 | 1.56 ± 0.90 | *0.234* | 0.132 | 31.20% |

| | | | | | | |
|---|---|---|---|---|---|---|
| | | (-7.24%) | (-10.26%) | | | |
| **10 cm above elbow – medial (subcutaneous thickness -cm)[a]** | 1.77 [1.28 - 2.02] | 1.53 [1.15 - 1.80] | 1.51 [0.93 - 1.65] | | | |
| **10 cm above elbow – posterior (subcutaneous thickness -cm)** | 2.50 ± 0.91 | 2.21 ± 0.83 (-10.40%) | 2.24 ± 0.87 (-9.77%) | *0.052* | 0.255 | 98.36% |
| | 2.33 [2.10 - 3.24] | 2.10 [1.94 - 2.41] | 2.21 [1.78 - 2.69] | | | |
| **10 cm above elbow – posterior (muscle thickness -cm)** | 1.63 ± 0.33 | 1.65 ± 0.22 (+2.94%) | 1.71 ± 0.31 (+7.35%) | *0.569* | 0.055 | 35.01% |
| | 1.67 [1.37 - 1.77] | 1.76 [1.47 - 1.82] | 1.77 [1.51 - 1.90] | | | |
| **10 cm above elbow – lateral (subcutaneous thickness -cm)** | 1.93 ± 0.31 | 1.77 ± 0.61 (-7.02%) | 1.74 ± 0.56 (-6.82%) | *0.201* | 0.148 | 80.88% |
| | 1.82 [1.36 - 2.50] | 1.60 [1.30 - 2.10] | 1.59 [1.36 - 2.10] | | | |
| **SELF-REPORTED QUESTIONNAIRES** | | | | | | |
| **Pain (NRS - points)[a]** | 2.64 ± 2.50 | 1.73 ± 2.10* (-36.36%) | 1.27 ± 2.00 (-40.91%) | ***0.038*** | 0.298 | 62.50% |
| | 2.00 [1.00 - 3.50] | 1.00 [0.00 - 2.50] | 0.00 [0.00 - 2.50] | | | |
| **DASH (score)** | 20.61 ± 13.29 | 13.94 ± 9.68 (-21.65%) | 17.50 ± 14.84 (-14.51%) | *0.110* | 0.198 | 92.72 % |
| | 18.33 [12.08 - 25.83] | 14.17 [5.42 - 20.83] | 16.67 [5.00 - 24.58] | | | |
| **FACT-B+4 physical well-being (score)** | 21.36 ± 2.50 | 23.82 ± 1.78 (+12.26%) | 21.91 ± 4.83 (+2.79%) | *0.112* | 0.220 | 95.56% |
| | 21.00 [19.50 - 22.50] | 24.00 [22.00 - 25.00] | 23.00 [19.50 - 25.50] | | | |

| | | | | | | |
|---|---|---|---|---|---|---|
| **FACT-B+4 social well-being (score)** | 21.00 ± 4.40 | 20.27 ± 3.77 (-2.10%) | 20.64 ± 4.97 (-2.00%) | *0.697* | 0.035 | 23.63% |
| | 22.00 [17.50 - 24.00] | 21.00 [17.50 - 23.50] | 23.00 [18.00 - 23.50] | | | |
| **FACT-B+4 emotional well-being (score)[a]** | 15.64 ± 3.98 | 17.18 ± 3.87* (+11.41%) | 15.45 ± 3.83 (-0.12%) | ***0.039*** | 0.295 | 62.00% |
| | 16.00 [13.50 - 18.50] | 18.00 [17.00 - 19.50] | 16.00 [13.50 - 18.00] | | | |
| **FCAT-B+4 functional well-being (score)** | 19.18 ± 4.07 | 20.82 ± 2.64 (+11.12%) | 20.36 ± 3.56 (+7.32%) | *0.052* | 0.256 | 98.36% |
| | 20.00 [16.00 - 22.00] | 21.00 [19.00 - 23.00] | 21.00 [18.00 - 22.50] | | | |
| **FACT-B+4 breast cancer subscale (score)** | 19.82 ± 3.89 | 24.82 ± 5.62 (+31.63%) | 22.36 ± 6.79 (+17.58%) | *0.100* | 0.233 | 98.24% |
| | 19.00 [17.00 - 23.00] | 25.00 [22.50 - 28.00] | 22.00 [17.50 - 27.50] | | | |
| **FACT-B+4 arm subscale (score)** | 13.82 ± 4.64 | 15.82 ± 3.43 (+22.65%) | 15.27 ± 3.98 (+14.33%) | ***0.016*** | 0.339 | +99% |
| | 16.00 [10.00 - 18.00] | 15.00 [13.50 - 19.00] | 15.00 [13.00 - 18.50] | | | |
| **FACT-B total (score)** | 94.45 ± 8.19 | 103.73 ± 9.90* (+9.99%) | 98.27 ± 15.71 (+3.80%) | ***0.011*** | 0.363 | +99% |
| | 94.00 [91.00 - 100.00] | 106.00 [99.50 - 110.00] | 104.00 [88.50 - 107.00] | | | |
| **FACT-B+4 total (score)** | 108.27 ± 10.43 | 119.55 ± 11.09* (+10.67%) | 113.55 ± 17.49 (+4.64%) | ***0.003*** | 0.434 | +99% |
| | 109.00 [101.00 - 117.50] | 120.00 [110.50 - 128.00] | 119.00 [101.50 - 124.00] | | | |

| | | | | | | |
|---|---|---|---|---|---|---|
| **Mean RR (ms)** | 886.85 ± 120.56 | 836.22 ± 113.08 (-5.43%) | 884.89 ± 135.82 (-0.02%) | *0.069* | 0.235 | 96.93% |
| | 908.07 [803.77 - 929.07] | 831.78 [741.68 - 927.97] | 832.75 [776.63 - 1028.90] | | | |
| **SDNN (ms)[a]** | 56.38 ± 70.29 | 38.65 ± 20.93 (-3.34%) | 33.49 ± 8.80 (-7.17%) | *0.529* | 0.058 | 15.80% |
| | 37.27 [31.81 - 44.96] | 33.16 [26.38 - 40.61] | 34.55 [26.82 - 40.33] | | | |
| **RMSSD (ms)[a]** | 46.08 ± 48.93 | 41.18 ± 32.45 (+9.41%) | 35.63 ± 13.50 (+12.56%) | *0.695* | 0.033 | 10.90% |
| | 30.71 [26.43 - 42.41] | 30.47 [18.68 - 51.16] | 36.35 [23.60 - 48.16] | | | |
| **LF FFT Power (log)** | 6.89 ± 1.80 | 6.14 ± 0.96 (-8.00%) | 6.03 ± 0.59 (-8.39%) | *0.123* | 0.209 | 94.28% |
| | 6.75 [5.97 - 7.20] | 6.08 [5.47 - 6.88] | 5.97 [5.64 - 6.39] | | | |
| **HF FFT Power (log)** | 5.94 ± 1.52 | 5.77 ± 1.57 (-2.01%) | 5.89 ± 0.65 (+3.51%) | *0.905* | 0.010 | 9.54% |
| | 5.49 [5.28 - 6.37] | 5.88 [4.73 - 6.66] | 5.87 [5.50 - 6.44] | | | |

[a] *Analyzed through non - parametric test. Friedman signed-rank test (for three time points) and Wilcoxon signed-rank test (for two time points).*

*Bold numbers show statistically significant differences: * p<0.05 compared to baseline; ** p≤0.001 compared to baseline, + p<0.05 compared to post-intervention.*

Abbreviations. BMI, Body mass index; cm, centimeters; DASH, Disabilities of the Arm, Shoulder, and Hand; SBP, Systolic blood pressure; DBP, Diastolic blood pressure; FACT-B+4, Functional Assessment of Cancer Therapy-Breast plus 4; FFT, Fast fournier transformation; HF, High Frequency; kg, Kilogram; LF, Low Frequency; m, Meters; Min, Minute; ml, milliliters; mmHg, millimeters of mercury; ms$^2$, Milliseconds squared; MVPA, Moderate-to-vigorous physical activity; N, Newton; NRS, Numerical Rating Scale; SD, Standard deviation; RR, Intervals between R-wave peaks; SDNN, Standard deviation of NN (Normal-to-Normal) intervals; RMSSD, Root mean square of the successive differences.

**Supplementary file S5**. Baseline, post-intervention and follow-up protein expression levels (NPX) values (n = 10 breast cancer survivors).

| Protein | Baseline | Post-intervention | Follow-up | Time | Effect size | Post hoc statistical Power |
|---|---|---|---|---|---|---|
| | Mean±SD<br>Median [Q1 - Q3] | Mean±SD<br>Median [Q1 - Q3] | Mean±SD<br>Median [Q1 - Q3] | *p-value* | (partial eta-squared or and Kendall's W) | |
| **ADA** | 5.68 ± 0.20 | 5.63 ± 0.26 | 5.32 ± 0.28 | ***<0.001*** | *0.714* | +99% |
| | 5.64 [5.56 - 5.78] | 5.67 [5.36 - 5.87] | 5.36 [5.21 - 5.5] | | | |
| **AXIN1** | 1.55 ± 0.35 | 1.31 ± 0.25 | 0.43 ± 0.24*+ | ***<0.001*** | *0.879* | +99% |
| | 1.59 [1.38 - 1.77] | 1.36 [1.30 - 1.43] | 0.38 [0.29 - 0.64] | | | |
| **CASP-8** | 3.32 ± 0.37 | 3.06 ± 0.31 | 2.02 ± 0.25*+ | ***<0.001*** | *0.944* | +99% |
| | 3.24 [3.13 - 3.57] | 3.06 [2.92 - 3.29] | 2.02 [1.90 - 2.13] | | | |
| **CCL3** | 6.55 ± 0.42 | 6.55 ± 0.55 | 5.70 ± 0.55*+ | ***<0.001*** | *0.816* | +99% |
| | 6.50 [6.20 - 6.74] | 6.57 [6.27 - 7.01] | 5.86 [5.27 - 6.14] | | | |
| **CCL4** | 6.79 ± 0.47 | 6.76 ± 0.55 | 6.23 ± 0.47*+ | ***<0.001*** | *0.750* | +99% |
| | 6.74 [6.47 - 6.92] | 6.83 [6.27 - 7.08] | 6.21 [6.02 - 6.54] | | | |
| **CCL11** | 7.62 ± 0.47 | 7.52 ± 0.38 | 7.11 ± 0.39* | ***<0.001*** | *0.716* | +99% |
| | 7.56 [7.36 - 7.9] | 7.53 [7.18 - 7.72] | 7.09 [6.94 - 7.2] | | | |
| **CCL19**[a] | 10.87 ± 1.36 | 11.04 ± 1.29 | 10.54 ± 1.47 | *0.006* | *0.520* | 83.12% |
| | 10.52 [10.07 - 11.06] | 10.86 [10.44 - 11.17] | 10.35 [9.68 - 10.65] | | | |
| **CCL20**[a] | 5.75 ± 1.30 | 6.29 ± 1.02 | 5.52 ± 1.65 | *0.020* | *0.390* | 70.59% |

| | | | | | | |
|---|---|---|---|---|---|---|
| | 5.67 [4.97 - 6.06] | 6.41 [5.51 - 6.75] | 5.00 [4.50 - 5.93] | | | |
| **CCL23** | 10.96 ± 0.33 | 11.08 ± 0.36 | 11.03 ± 0.30 | *0.569* | *0.061* | 35.04% |
| | 10.92 [10.72 - 11.14] | 11.10 [11.02 - 11.20] | 11.02 [10.95 - 11.11] | | | |
| **CCL25** | 6.41 ± 0.61 | 6.42 ± 0.54 | 5.82 ± 0.59 | *0.003* | ***0.603*** | +99% |
| | 6.32 [5.89 - 6.86] | 6.27 [6.01 - 6.85] | 5.76 [5.4 - 6.11] | | | |
| **CCL28** | 3.18 ± 0.30 | 3.17 ± 0.29 | 2.53 ± 0.31*+ | ***<0.001*** | ***0.880*** | +99% |
| | 3.09 [3.00 - 3.33] | 3.12 [3.00 - 3.30] | 2.47 [2.35 - 2.65] | | | |
| **CD5** | 5.62 ± 0.25 | 5.53 ± 0.29 | 5.27 ± 0.27 | ***<0.001*** | *0.636* | +99% |
| | 5.61 [5.50 - 5.80] | 5.59 [5.46 - 5.73] | 5.27 [5.10 - 5.42] | | | |
| **CD6** | 6.52 ± 0.31 | 6.41 ± 0.31 | 5.58 ± 0.36*+ | ***<0.001*** | *0.873* | +99% |
| | 6.48 [6.29 - 6.72] | 6.48 [6.19 - 6.65] | 5.60 [5.37 - 5.89] | | | |
| **CD8A** | 7.23 ± 0.63 | 7.19 ± 0.73 | 7.29 ± 0.58 | *0.591* | *0.057* | 33.87% |
| | 7.16 [6.76 - 7.63] | 7.02 [6.83 - 7.83] | 7.30 [6.98 - 7.65] | | | |
| **CD40** | 11.83 ± 0.20 | 11.70 ± 0.21 | 11.29 ± 0.22*+ | ***<0.001*** | *0.888* | +99% |
| | 11.84 [11.73 - 11.88] | 11.72 [11.53 - 11.8] | 11.28 [11.1 - 11.45] | | | |
| **CD244** | 6.72 ± 0.29 | 6.58 ± 0.35 | 6.81 ± 0.25 | *0.053* | *0.279* | 98.23% |
| | 6.69 [6.59 - 6.82] | 6.63 [6.40 - 6.79] | 6.82 [6.61 - 6.96] | | | |
| **CDCP1** | 4.65 ± 0.42 | 4.51 ± 0.42 | 3.59 ± 0.46*+ | ***<0.001*** | *0.945* | +99% |
| | 4.60 [4.53 - 4.9] | 4.50 [4.24 - 4.62] | 3.55 [3.44 - 3.72] | | | |

| | | | | | | |
|---|---|---|---|---|---|---|
| **CSF-1** | 10.38 ± 0.11 | 10.28 ± 0.15 | 10.06 ± 0.15* | ***<0.001*** | *0.656* | +99% |
| | 10.39 [10.35 - 10.44] | 10.27 [10.19 - 10.37] | 10.05 [9.99 - 10.11] | | | |
| **CST5** | 7.23 ± 0.41 | 7.20 ± 0.54 | 6.91 ± 0.48 | *0.087* | *0.272* | 97.90% |
| | 7.19 [7.04 - 7.47] | 7.14 [6.83 - 7.57] | 6.84 [6.63 - 7.24] | | | |
| **CX3CL1** | 4.42 ± 0.36 | 4.35 ± 0.31 | 3.17 ± 0.37*+ | ***<0.001*** | *0.953* | +99% |
| | 4.38 [4.26 - 4.6] | 4.36 [4.26 - 4.52] | 3.12 [2.99 - 3.34] | | | |
| **CXCL1** | 9.94 ± 1.11 | 10.14 ± 1.18 | 10.16 ± 0.62 | *0.651* | *0.047* | 27.40% |
| | 10.07 [9.36 - 10.73] | 10.55 [9.22 - 11.01] | 10.25 [9.96 - 10.51] | | | |
| **CXCL5** | 10.01 ± 1.76 | 10.51 ± 1.82 | 11.66 ± 1.30 | *0.001* | *0.568* | +99% |
| | 10.18 [9.59 - 10.86] | 10.52 [9.02 - 11.71] | 11.39 [10.8 - 12.35] | | | |
| **CXCL6** | 7.25 ± 0.56 | 7.64 ± 0.58 | 7.86 ± 0.49 | *0.013* | *0.385* | +99% |
| | 7.38 [6.77 - 7.55] | 7.09 [7.24 - 8.00] | 8.06 [7.52 - 8.18] | | | |
| **CXCL9**[a] | 7.34 ± 0.54 | 6.98 ± 0.39 | 7.65 ± 1.07 | *0.150* | *0.190* | 39.70% |
| | 7.22 [6.92 - 7.73] | 7.09 [6.73 - 7.18] | 7.38 [7.07 - 8.02] | | | |
| **CXCL10**[a] | 9.53 ± 0.30 | 9.66 ± 0.34 | 8.01 ± 0.37 | ***<0.001*** | *0.790* | 95.44% |
| | 9.62 [9.47 - 9.69] | 9.67 [9.43 - 9.92] | 8.12 [7.77 - 8.23] | | | |
| **CXCL11** | 7.48 ± 0.60 | 7.51 ± 0.61 | 8.20 ± 0.84 | ***<0.001*** | *0.583* | +99% |
| | 7.57 [7.19 - 7.67] | 7.48 [7.09 - 7.6] | 8.07 [7.71 - 8.31] | | | |
| **DNER** | 8.32 ± 0.22 | 8.27 ± 0.28 | 8.88 ± 0.26*+ | ***<0.001*** | *0.812* | +99% |

| | | | | | | |
|---|---|---|---|---|---|---|
| | 8.31 [8.21 - 8.47] | 8.27 [8.13 - 8.50] | 8.89 [8.76 - 9.05] | | | |
| **EN-RAGE** | 3.75 ± 0.68 | 3.37 ± 0.66 | 2.14 ± 0.51*+ | ***<0.001*** | *0.826* | +99% |
| | 3.88 [3.13 - 4.28] | 3.37 [2.99 - 3.66] | 1.96 [1.77 - 2.50] | | | |
| **FGF-19** | 7.97 ± 0.87 | 7.90 ± 1.18 | 7.70 ± 1.04 | *0.518* | *0.070* | 39.81% |
| | 8.07 [7.28 - 8.69] | 7.98 [7.11 - 8.79] | 7.67 [7.00 - 8.19] | | | |
| **FGF-21** | 4.08 ± 0.77 | 4.48 ± 1.05 | 3.99 ± 1.17 | *0.428* | *0.090* | 50.37% |
| | 4.14 [3.58 - 4.53] | 4.69 [4.25 - 5.01] | 4.18 [2.95 - 4.82] | | | |
| **FGF-23** | 2.24 ± 0.35 | 2.13 ± 0.58 | 2.08 ± 0.40 | *0.373* | *0.104* | 57.59% |
| | 2.26 [2.12 - 2.36] | 2.13 [1.84 - 2.31] | 1.99 [1.90 - 2.28] | | | |
| **Flt3L** | 9.10 ± 0.33 | 8.89 ± 0.48 | 9.31 ± 0.31 | *0.018* | *0.359* | +99% |
| | 8.97 [8.86 - 9.32] | 8.90 [8.69 - 9.14] | 9.32 [9.05 - 9.49] | | | |
| **GDNF** | 1.11 ± 0.26 | 0.99 ± 0.37 | 1.55 ± 0.29 | *0.002* | *0.492* | +99% |
| | 1.07 [0.90 - 1.23] | 1.02 [0.91 - 1.23] | 1.50 [1.35 - 1.68] | | | |
| **HGF** | 9.30 ± 0.32 | 9.21 ± 0.37 | 8.99 ± 0.30 | *0.018* | *0.428* | +99% |
| | 9.26 [9.00 - 9.56] | 9.16 [8.95 - 9.44] | 8.96 [8.73 - 9.21] | | | |
| **IFN-gamma** | 7.09 ± 0.43 | 6.93 ± 0.38 | 6.88 ± 0.98 | *0.644* | *0.031* | 19.06% |
| | 6.98 [6.87 - 7.32] | 6.94 [6.7 - 7.12] | 6.49 [6.19 - 7.51] | | | |
| **IL-6[a]** | 3.46 ± 1.29 | 3.02 ± 1.51 | 2.99 ± 0.75 | *0.150* | *0.190* | 39.70% |
| | 2.97 [2.51 - 4.68] | 2.47 [2.09 - 3.38] | 2.87 [2.35 - 3.60] | | | |

| | | | | | | |
|---|---|---|---|---|---|---|
| **IL-7** | 0.92 ± 0.38 | 1.11 ± 0.43 | 1.19 ± 0.30 | *0.272* | *0.135* | 71.16% |
| | 0.86 [0.6 - 1.29] | 1.07 [0.89 - 1.28] | 1.08 [0.95 - 1.46] | | | |
| **IL-8** | 7.47 ± 0.50 | 7.44 ± 0.42 | 6.69 ± 0.52+ | ***<0.001*** | ***0.710*** | +99% |
| | 7.37 [7.21 - 7.62] | 7.43 [7.36 - 7.64] | 6.86 [6.35 - 7.01] | | | |
| **IL-10** | 3.61 ± 0.43 | 3.68 ± 0.74 | 3.52 ± 0.58 | *0.776* | *0.028* | 17.57% |
| | 3.58 [3.36 - 3.89] | 3.59 [3.08 - 3.93] | 3.48 [3.1 - 3.96] | | | |
| **IL-18** | 8.48 ± 0.55 | 8.40 ± 0.62 | 8.96 ± 0.67 | *0.079* | *0.291* | 98.75% |
| | 8.42 [8.01 - 8.84] | 8.29 [8.01 - 8.91] | 8.90 [8.52 - 9.30] | | | |
| **IL-10RA**[a] | 1.57 ± 1.52 | 1.52 ± 1.57 | 1.35 ± 1.33 | *0.150* | *0.190* | 39.70% |
| | 0.71 [0.43 - 2.52] | 0.93 [0.34 - 2.74] | 0.69 [0.46 - 1.94] | | | |
| **IL-10RB** | 6.52 ± 0.24 | 6.42 ± 0.27 | 6.66 ± 0.20 | *0.019* | *0.358* | +99% |
| | 6.62 [6.35 - 6.70] | 6.47 [6.31 - 6.59] | 6.70 [6.65 - 6.77] | | | |
| **IL-12B** | 6.53 ± 0.54 | 6.47 ± 0.6 | 6.42 ± 0.53 | *0.838* | *0.019* | 13.13% |
| | 6.37 [6.20 - 7.00] | 6.26 [6.01 - 7.00] | 6.28 [6.01 - 6.86] | | | |
| **IL-15RA** | 0.86 ± 0.22 | 0.71 ± 0.25 | 0.31 ± 0.29 | ***<0.001*** | *0.754* | +99% |
| | 0.84 [0.81 - 0.94] | 0.64 [0.5 - 0.93] | 0.34 [0.18 - 0.53] | | | |
| **IL-17C** | 3.20 ± 0.74 | 3.50 ± 0.68 | 2.87 ± 0.78 | *0.001* | *0.539* | +99% |
| | 3.00 [2.60 - 3.79] | 3.37 [3.00- 4.07] | 2.65 [2.27 - 3.60] | | | |
| **IL-18R1** | 8.49 ± 0.41 | 8.36 ± 0.43 | 8.52 ± 0.38 | *0.071* | *0.254* | 98.62% |

| | | | | | | |
|---|---|---|---|---|---|---|
| | 8.41 [8.13 - 8.85] | 8.34 [7.97 - 8.74] | 8.57 [8.19 - 8.82] | | | |
| **LAP TGF -beta-1** | 6.60 ± 0.24 | 6.33 ± 0.21 | 6.43 ± 0.21 | *0.006* | *0.439* | +99% |
| | 6.71 [6.51 - 6.75] | 6.3 [6.22 - 6.53] | 6.42 [6.35 - 6.55] | | | |
| **LIF-R** | 5.05 ± 0.16 | 4.92 ± 0.16 | 4.60 ± 0.09*+ | ***<0.001*** | *0.802* | +99% |
| | 5.02 [4.95 - 5.17] | 4.95 [4.91 - 4.98] | 4.62 [4.55 - 4.64] | | | |
| **MCP-1**[a] | 12.04 ± 0.56 | 11.93 ± 0.38 | 11.31 ± 0.43 | *0.001* | *0.760* | +99% |
| | 11.95 [11.64 - 12.27] | 12.02 [11.64 - 12.15] | 11.17 [11.12 - 11.53] | | | |
| **MCP-2** | 8.22 ± 0.42 | 8.21 ± 0.31 | 8.78 ± 0.40*+ | ***<0.001*** | *0.849* | +99% |
| | 8.28 [7.90 - 8.50] | 8.2 [7.94 - 8.45] | 8.8 [8.58 - 8.89] | | | |
| **MCP-3** | 1.98 ± 0.72 | 2.01 ± 0.60 | 2.23 ± 0.55 | *0.155* | *0.187* | 87.27% |
| | 2.00 [1.31 - 2.42] | 1.83 [1.55 - 2.49] | 2.36 [1.86 - 2.61] | | | |
| **MCP-4** | 14.91 ± 0.65 | 14.85 ± 0.52 | 15.14 ± 0.64 | *0.015* | *0.372* | +99% |
| | 14.84 [14.56 - 15.10] | 14.94 [14.61 - 15.16] | 15.10 [14.87 - 15.41] | | | |
| **MMP-1**[a] | 12.33 ± 0.95 | 12.33 ± 0.72 | 11.70 ± 0.71 | *0.014* | *0.430* | 75.05% |
| | 12.76 [11.8 - 12.98] | 12.12 [11.71 - 13.02] | 11.73 [11.47 - 12.23] | | | |
| **MMP-10**[a] | 9.15 ± 0.74 | 8.86 ± 0.39 | 9.11 ± 0.42 | *0.122* | *0.210* | 43.36% |
| | 8.94 [8.74 - 9.27] | 8.84 [8.59 - 9.00] | 9.10 [8.79 - 9.31] | | | |
| **NT-3** | 3.91 ± 0.23 | 3.68 ± 0.36 | 2.31 ± 0.43*+ | ***<0.001*** | *0.840* | +99% |
| | 3.85 [3.75 - 4.08] | 3.74 [3.52 - 3.85] | 2.25 [2.01 - 2.59] | | | |

| | | | | | | |
|---|---|---|---|---|---|---|
| **OPG** | 10.52 ± 0.33 | 10.43 ± 0.37 | 10.11 ± 0.35*+ | ***<0.001*** | *0.841* | +99% |
| | 10.42 [10.37 - 10.77] | 10.46 [10.22 - 10.6] | 10.07 [9.93 - 10.25] | | | |
| **OSM** | 5.27 ± 1.09 | 4.99 ± 1.2 | 4.10 ± 0.91 | *0.002* | *0.508* | +99% |
| | 5.12 [4.49 - 6.08] | 4.60 [4.07 - 5.94] | 4.00 [3.40 - 4.77] | | | |
| **PD-L1** | 7.12 ± 0.25 | 7.01 ± 0.29 | 6.68 ± 0.23*+ | ***<0.001*** | *0.714* | +99% |
| | 7.07 [7.02 - 7.18] | 7.03 [6.79 - 7.18] | 6.65 [6.51 - 6.82] | | | |
| **4E-BP1** | 5.24 ± 0.64 | 5.29 ± 0.49 | 5.36 ± 0.52 | *0.748* | *0.018* | 12.64% |
| | 5.06 [4.79 - 5.62] | 5.32 [5.13 - 5.42] | 5.32 [5.01 - 5.73] | | | |
| **SCF** | 9.12 ± 0.47 | 9.08 ± 0.49 | 9.00 ± 0.52 | *0.616* | *0.031* | 19.06% |
| | 9.28 [8.91 - 9.46] | 9.27 [8.82 - 9.46] | 9.15 [8.68 - 9.36] | | | |
| **SIRT2** | 2.63 ± 0.56 | 2.65 ± 0.40 | 2.23 ± 0.34 | *0.062* | *0.311* | +99% |
| | 2.70 [2.14 - 2.95] | 2.55 [2.42 - 2.79] | 2.24 [2.00 - 2.42] | | | |
| **SLAMF1** | 2.77 ± 0.26 | 2.65 ± 0.4 | 2.65 ± 0.25 | *0.346* | *0.111* | 60.74% |
| | 2.80 [2.67 - 2.94] | 2.64 [2.41 - 2.97] | 2.57 [2.49 - 2.73] | | | |
| **ST1A1** | 3.53 ± 0.46 | 3.54 ± 0.48 | 2.95 ± 0.59 | *0.027* | *0.405* | +99% |
| | 3.50 [3.29 - 3.88] | 3.62 [3.24 - 3.93] | 2.83 [2.71 - 3.22] | | | |
| **STAMBP** | 5.46 ± 0.40 | 5.44 ± 0.35 | 4.28 ± 0.32*+ | ***<0.001*** | *0.856* | +99% |
| | 5.49 [5.16 - 5.71] | 5.44 [5.19 - 5.63] | 4.32 [4.08 - 4.51] | | | |
| **TGFalpha** | 1.66 ± 0.41 | 1.63 ± 0.68 | 1.87 ± 0.28 | *0.183* | *0.172* | 83.52% |

| | | | | | | |
|---|---|---|---|---|---|---|
| | 1.59 [1.38 - 1.78] | 1.51 [1.33 - 1.77] | 1.74 [1.65 - 2.01] | | | |
| **TNF** | 4.30 ± 0.33 | 4.41 ± 0.39 | 3.37 ± 0.42*+ | ***<0.001*** | *0.929* | +99% |
| | 4.22 [4.12 - 4.61] | 4.38 [4.17 - 4.74] | 3.26 [3.02 - 3.71] | | | |
| **TNFB** | 3.79 ± 0.33 | 3.66 ± 0.28 | 3.45 ± 0.23* | ***<0.001*** | *0.676* | +99% |
| | 3.78 [3.67 - 3.93] | 3.67 [3.57 - 3.83] | 3.51 [3.33 - 3.55] | | | |
| **TNFRSF9** | 6.58 ± 0.31 | 6.44 ± 0.38 | 5.41 ± 0.35*+ | ***<0.001*** | *0.940* | +99% |
| | 6.61 [6.33 - 6.71] | 6.50 [6.23 - 6.64] | 5.33 [5.16 - 5.54] | | | |
| **TNFSF14**[a] | 4.35 ± 0.43 | 4.25 ± 0.51 | 3.55 ± 0.43 | *0.002* | *0.630* | 89.87% |
| | 4.24 [4.01 - 4.72] | 4.09 [3.87 - 4.74] | 3.37 [3.26 - 3.82] | | | |
| **TRAIL** | 9.22 ± 0.31 | 9.11 ± 0.39 | 7.98 ± 0.39*+ | ***<0.001*** | *0.894* | +99% |
| | 9.34 [9.03 - 9.40] | 9.14 [9.02 - 9.39] | 8.09 [7.79 - 8.22] | | | |
| **TRANCE** | 5.91 ± 0.60 | 5.82 ± 0.67 | 5.45 ± 0.66 | *0.067* | *0.260* | 97.19% |
| | 5.87 [5.50 - 6.31] | 5.96 [5.37 - 6.23] | 5.46 [4.91 - 5.92] | | | |
| **TWEAK** | 9.98 ± 0.27 | 9.85 ± 0.28 | 9.10 ± 0.27*+ | ***<0.001*** | *0.904* | +99% |
| | 0.85 [0.45 - 1.02] | 9.83 [9.68 - 10.10] | 9.10 [8.97 - 9.26] | | | |
| **uPA** | 10.90 ± 0.35 | 10.90 ± 0.39 | 10.64 ± 0.35 | *0.010* | *0.401* | +99% |
| | 10.98 [10.62 - 11.14] | 11.04 [10.72 - 11.10] | 10.64 [10.53 - 10.74] | | | |
| **VEGF-A** | 11.00 ± 0.24 | 10.92 ± 0.19 | 10.91 ± 0.20 | *0.258* | *0.140* | 73.21% |
| | 11.03 [10.80 - 11.18] | 10.95 [10.85 - 11.00] | 10.94 [10.85 - 11.02] | | | |

[a] *Analyzed through non-parametric tests. Friedman signed-rank test (for three time points) and Wilcoxon signed-rank test (for two time points).*

*Bold numbers show statistically significant differences: * $p<0.001$* compared to baseline; + *$p<0.001$* compared to post-intervention

Abbreviations: 4E-BP1, Eukaryotic translation initiation factor 4E-binding protein 1; ADA, Adenosine deaminase; AXIN1, Axin-1; CASP-8, Caspase-8; CCL3, C-C motif chemokine 3; CCL4, C-C motif chemokine 4; CCL11, Eotaxin; CCL19, C-C motif chemokine 19; CCL20, C-C motif chemokine 20; CCL23, C-C motif chemokine 23; CCL25, C-C motif chemokine 25; CCL28, C-C motif chemokine 28; CD5, T-cell surface glycoprotein CD5; CD6, T cell surface glycoprotein CD6 isoform; CD40, CD40L receptor; CD244, Natural killer cell receptor 2B4; CD8A, T-cell surface glycoprotein; CDCP1, CUB domain-containing protein 1; CSF-1, Macrophage colony-stimulating factor 1; CST5, Cystatin D; CX3CL1, Fractalkine; CXCL1, C-X-C motif chemokine 1; CXCL5, C-X-C motif chemokine 5; CXCL6, C-X-C motif chemokine 6; CXCL9, C-X-C motif chemokine 9; CXCL10, C-X-C motif chemokine 10; CXCL11, C-X-C motif chemokine 11; DNER, Delta and Notch-like epidermal growth factor-related receptor; EN-RAGE, Protein S100-A12; FGF-19, Fibroblast growth factor 19; FGF-21, Fibroblast growth factor 21; FGF-23, Fibroblast growth factor 23; Flt3L, Fms-related tyrosine kinase 3 ligand; GDNF, Glial cell line-derived neurotrophic factor; HGF, Hepatocyte growth factor; IFN-gamma, Interferon gamma; IL-6, interleukin 6; IL-7, Interleukin-7; IL-8, Interleukin 8; IL10, Interleukin-10; IL-18, interleukin 18; IL-10RA, Interleukin-10 receptor subunit alpha; IL-10RB, Interleukin-10 receptor subunit beta; IL-12B, Interleukin-12 subunit beta; IL-15RA, Interleukin-15 receptor subunit alpha; IL-17C, Interleukin-17C; IL-18R1, Interleukin-18 receptor 1; IL-20RA, Interleukin 20 receptor subunit alpha; LAP TGF-beta – 1, Transforming growth factor beta-1 proprotein; LIF-R, Leukemia inhibitory factor receptor; MCP-1, Monocyte chemotactic protein 1; MCP-2, Monocyte chemotactic protein 2; MCP-3, Monocyte chemotactic protein 3; MCP-4, Monocyte chemotactic protein 4; MMP-1, Matrix metalloproteinase-1; MMP-10, Matrix metalloproteinase-10; NPX, Normalized Protein eXpression; NT-3, Neurotrophin-3; OSM, Oncostatin-M; OPG, Osteoprotegerin; PD-L1, Programmed cell death 1 ligand 1; SCF, Stem cell factor; SD, Standard deviation; SLAMF1, Signaling lymphocytic activation molecule; SIRT2, SIR2-like protein 2; STAMBP, STAM-binding protein; ST1A1, Sulfotransferase 1A1; TGF-alpha, Transforming growth factor alpha; TNF, Tumor necrosis factor; TNFB, TNF-beta; TNFRSF9, Tumor necrosis factor receptor superfamily member 9; TNFSF14, Tumor necrosis factor ligand superfamily member 14; TRAIL, TNF-related apoptosis-inducing ligand; TRANCE, TNF-related activation-induced cytokine; TWEAK, Tumor necrosis factor (Ligand) superfamily member 12; uPA, Urokinase-type plasminogen activator; VEGF-A, Vascular endothelial growth factor A, long form,

**Supplementary file S6.** Estimated effect size [Cohen's d and 95% confidence interval and the rank biserial correlation (r) and 95% confidence interval].

| Outcome | Post-intervention - baseline | Follow-up - baseline | Follow-up – post-intervention |
|---|---|---|---|
| Unilateral chest press (affected arm) | 1.66 (0.75, 2.57) | 0.82 (0.14, 1.51) | -1.95 (-2.95, -0.94) |
| Unilateral chest press (unaffected arm) | 1.25 (0.46, 2.03) | 0.64 (-0.01, 1.29) | -0.78 (-1.45, -0.11) |
| Unilateral row (affected arm) | 1.34 (0.52, 2.15) | 0.88 (0.19, 1.58) | -0.99 (-1.71, -0.27) |
| Unilateral row (unaffected arm) | 1.70 (0.77, 2.62) | 0.98 (0.26, 1.70) | -0.95 (-1.66, -0.23) |
| Bilateral squat | 0.67 (0.02, 1.33) | 0.35 (-0.25, 0.96) | -0.80 (-1.47, -0.12) |
| Handgrip strength (affected arm)[a] | -0.68 (-0.91, -0.14) | -0.36 (-0.79, 0.30) | -0.55 (-0.86, 0.08) |
| Handgrip strength (unaffected arm) | 0.83 (0.15, 1.52) | -0.11 (-0.71, 0.48) | -0.88 (-1.58, -0.18) |
| MVPA[a] | -0.52 (-0.85, 0.11) | -0.36 (-0.79, 0.30) | -0.09 (-0.66, 0.54) |
| $VO_{2peak}$ | 0.21 (-0.38, 0.81) | -0.20 (-0.80, 0.40) | -0.55 (-1.18, 0.09) |
| Arm volume (affected arm)[a] | -0.25 (-0.74, 0.41) | -0.31 (-0.77, 0.36) | -0.66 (-0.90, -0.09) |
| Arm volume (unaffected arm) | 0.29 (-0.31, 0.90) | 0.11 (-0.49, 0.70) | -0.12 (-0.72, 0.47) |
| Inter-arm volume difference | -0.04 (-0.63, 0.55) | 0.35 (-0.26, 0.96) | 0.54 (-0.09, 1.17) |
| Shoulder flexion (affected arm)[a] | 0.08 (-0.55, 0.65) | 0.05 (-0.56, 0.63) | 0.00 (-0.60, 0.60) |
| Shoulder flexion (unaffected arm)[a] | -0.25 (-0.74, 0.41) | 0.00 (-0.60, 0.60) | -0.28 (-0.75, 0.38) |
| Shoulder abduction (affected arm)[a] | 0.00 (-0.60, 0.60) | 0.11 (-0.53, 0.66) | -0.42 (-0.81, 0.25) |
| Shoulder abduction (unaffected arm)[a] | 0.00 (-0.60, 0.60) | 0.00 (-0.60, 0.60) | -0.58 (-0.87, 0.04) |
| BMI | -0.03 (-0.62, 0.57) | -0.07 (-0.67, 0.52) | -0.08 (-0.67, 0.52) |

| | | | |
|---|---|---|---|
| 10 cm below elbow – anterior (subcutaneous thickness) | -0.48 (-1.10, 0.15) | -0.61 (-1.26, 0.03) | 0.03 (-0.56, 0.63) |
| 10 cm below elbow – medial (subcutaneous thickness) | -0.61 (-1.25, 0.04) | -0.88 (-1.57, -0.18) | -0.42 (-1.03, 0.20) |
| 10 cm below elbow – medial (muscle thickness) | 0.01 (-0.58, 0.60) | -0.54 (-1.18, 0.09) | -0.53 (-1.16, 0.10) |
| 10 cm below elbow – posterior (subcutaneous thickness )[a] | -0.52 (-0.85, 0.11) | -0.39 (-0.80, 0.28) | -0.19 (-0.71, 0.46) |
| 10 cm below elbow – lateral (subcutaneous thickness )[a] | -0.50 (-0.84, 0.15) | -0.39 (-0.80, 0.28) | -0.13 (-0.68, 0.51) |
| 10 cm below elbow – lateral (muscle thickness ) | 0.40 (-0.22, 1.01) | 0.08 (-0.51, 0.67) | -0.49 (-1.11, 0.14) |
| 10 cm above elbow – anterior (subcutaneous thickness )[a] | -0.44 (-0.82, 0.21) | -0.09 (-0.66, 0.54) | -0.15 (-0.69, 0.50) |
| 10 cm above elbow – anterior (muscle thickness ) | 0.70 (0.04, 1.36) | 0.29 (-0.31, 0.89) | -0.19 (-0.79, 0.41) |
| 10 cm above elbow – medial (subcutaneous thickness)[a] | -0.71 (-0.92, -0.19) | -0.47 (-0.83, 0.18) | -0.07 (-0.64, 0.56) |
| 10 cm above elbow – posterior (subcutaneous thickness ) | -0.76 (-1.43, -0.09) | -0.52 (-1.15, 0.11) | 0.07 (-0.52, 0.66) |
| 10 cm above elbow – posterior (muscle thickness) | 0.09 (-0.50, 0.68) | 0.27 (-0.34, 0.87) | 0.24 (-0.36, 0.84) |
| 10 cm above elbow – lateral (subcutaneous thickness) | -0.71 (-1.37, -0.05) | -0.42 (-1.04, 0.20) | -0.09 (-0.68, 0.50) |
| Pain (NRS) | -1.30 (-2.10, -0.50) | -0.54 (-1.17, 0.10) | -0.19 (-0.79, 0.40) |
| DASH | -0.60 (-1.24, 0.04) | -0.30 (-0.90, 0.31) | 0.45 (-0.17, 1.07) |
| FACT-B+4 physical well-being | 1.40 (0.57, 2.23) | 0.13 (-0.47, 0.72) | -0.45 (-1.07, 0.17) |
| FACT-B+4 social well-being | -0.27 (-0.87, 0.33) | -0.13 (-0.73, 0.46) | 0.12 (-0.47, 0.72) |
| FACT-B+4 emotional well-being[a] | -0.20 (-0.71, 0.45) | -0.21 (-0.72, 0.44) | -0.55 (-0.86, 0.08) |
| FCAT-B+4 functional well-being | 0.62 (-0.03, 1.26) | 0.69 (0.03, 1.34) | -0.24 (-0.84, 0.36) |
| FACT-B+4 breast cancer subscale | 0.65 (0.00, 1.30) | 0.31 (-0.29, 0.92) | -0.75 (-1.42, -0.08) |

| | | | |
|---|---|---|---|
| FACT-B+4 arm subscale | 0.79 (0.11, 1.47) | 0.83 (0.14, 1.51) | -0.26 (-0.87, 0.34) |
| FACT-B total | 1.28 (0.49, 2.08) | 0.33 (-0.28, 0.93) | -0.70 (-1.36, -0.04) |
| FACT-B+4 total | 1.55 (0.68, 2.43) | 0.45 (-0.17, 1.08) | -0.64 (-1.29, 0.01) |
| Mean RR | -0.68 (-1.34, -0.03) | -0.02 (-0.61, 0.57) | 0.72 (0.06, 1.38) |
| SDNN[a] | -0.34 (-0.78, 0.33) | -0.36 (-0.79, 0.30) | -0.20 (-0.71, 0.45) |
| RMSSD[a] | -0.36 (-0.79, 0.30) | 0.01 (-0.59, 0.61) | -0.01 (-0.61, 0.59) |
| LF FFT Power | -0.55 (-1.18, 0.09) | -0.50 (-1.12, 0.13) | -0.14 (-0.73, 0.45) |
| HF FFT Power | -0.15 (-0.74, 0.45) | -0.03 (-0.62, 0.56) | 0.10 (-0.49, 0.69) |
| ADA | -0.31 (-1.03, 0.40) | -1.80 (-2.51, -1.08) | -1.66 (-2.38, -0.95) |
| AXIN1 | -0.57 (-1.28, 0.15) | -3.66 (-4.37, -2.94) | -4.05 (-4.76, -3.33) |
| CASP-8 | -0.95 (-1.66, -0.23) | -6.81 (-7.52, -6.09) | -3.75 (-4.46, -3.03) |
| CCL3 | 0.00 (-0.72, 0.71) | -2.10 (-2.81, -1.38) | -2.41 (-3.12, -1.69) |
| CCL4 | -0.06 (-0.78, 0.65) | -2.54 (-3.25, -1.82) | -2.35 (-3.06, -1.63) |
| CCL11 | -0.32 (-1.04, 0.40) | -2.37 (-3.09, -1.66) | -1.93 (-2.64, -1.21) |
| CCL19[a] | -0.90 (-0.98, -0.63) | -0.90 (-0.98, -0.63) | -0.90 (-0.98, -0.63) |
| CCL20[a] | -0.74 (-0.93, -0.21) | -0.29 (-0.78, 0.42) | -0.61 (-0.90, 0.03) |
| CCL23 | 0.47 (-0.24, 1.19) | 0.18 (-0.53, 0.90) | -0.13 (-0.84, 0.59) |
| CCL25 | 0.02 (-0.69, 0.74) | -1.11 (-1.82, -0.39) | -2.85 (-3.56, -2.13) |
| CCL28 | -0.03 (-0.75, 0.68) | -3.45 (-4.16, -2.73) | -3.14 (-3.86, -2.43) |

| | | | |
|---|---|---|---|
| CD5 | -0.39 (-1.11, 0.32) | -1.70 (-2.41, -0.98) | -1.51 (-2.22, -0.79) |
| CD6 | -0.33 (-1.04, 0.39) | -2.97 (-3.68, -2.25) | -3.93 (-4.65, -3.21) |
| CD8A | -0.14 (-0.86, 0.58) | 0.16 (-0.56, 0.87) | 0.44 (-0.28, 1.15) |
| CD40 | -0.90 (-1.62, -0.19) | -2.87 (-3.59, -2.16) | -4.01 (-4.73, -3.30) |
| CD244 | -0.58 (-1.29, 0.14) | 0.39 (-0.33, 1.10) | 0.66 (-0.06, 1.38) |
| CDCP1 | -0.67 (-1.38, 0.05) | -4.59 (-5.31, -3.88) | -5.60 (-6.31, -4.88) |
| CSF1 | -0.51 (-1.22, 0.21) | -1.51 (-2.22, -0.79) | -2.06 (-2.77, -1.34) |
| CST5 | -0.12 (-0.84, 0.59) | -0.68 (-1.40, 0.03) | -0.54 (-1.26, 0.17) |
| CX3CL1 | -0.40 (-1.11, 0.32) | -5.10 (-5.81, -4.38) | -4.56 (-5.27, -3.84) |
| CXCL1 | 0.27 (-0.45, 0.98) | 0.26 (-0.46, 0.97) | 0.02 (-0.70, 0.73) |
| CXCL5 | 0.47 (-0.24, 1.19) | 1.26 (0.55, 1.98) | 1.23 (0.52, 1.95) |
| CXCL6 | 0.84 (0.13, 1.56) | 1.07 (0.35, 1.78) | 0.32 (-0.39, 1.04) |
| CXCL9[a] | -0.29 (-0.78, 0.42) | -0.26 (-0.76, 0.44) | -0.71 (-0.93, -0.14) |
| CXCL10[a] | -0.23 (-0.75, 0.47) | -0.90 (-0.98, -0.63) | -0.90 (-0.98, -0.63) |
| CXCL11 | 0.06 (-0.65, 0.78) | 1.25 (0.53, 1.96) | 1.43 (0.71, 2.14) |
| DNER | -0.27 (-0.99, 0.44) | 1.99 (1.27, 2.70) | 2.40 (1.68, 3.11) |
| EN-RAGE | -0.54 (-1.25, 0.18) | -3.47 (-4.18, -2.75) | -2.31 (-3.02, -1.59) |
| FGF-19 | -0.07 (-0.79, 0.64) | -0.38 (-1.09, 0.34) | -0.32 (-1.04, 0.39) |
| FGF-21 | 0.35 (-0.36, 1.07) | -0.06 (-0.78, 0.65) | -0.45 (-1.17, 0.26) |

| | | | |
|---|---|---|---|
| FGF-23 | -0.31 (-1.02, 0.41) | -0.43 (-1.15, 0.28) | -0.16 (-0.87, 0.56) |
| Flt3L | -0.68 (-1.40, 0.04) | 0.52 (-0.19, 1.24) | 0.81 (0.10, 1.53) |
| GDNF | -0.32 (-1.04, 0.39) | 1.22 (0.51, 1.94) | 0.97 (0.25, 1.68) |
| HGF | -0.25 (-0.97, 0.46) | -1.12 (-1.83, -0.40) | -1.34 (-2.06, -0.63) |
| IFN-gamma | -0.42 (-1.14, 0.29) | -0.19 (-0.90, 0.53) | -0.04 (-0.76, 0.67) |
| IL-6[a] | -0.42 (-0.83, 0.29) | -0.26 (-0.76, 0.44) | -0.42 (-0.83, 0.29) |
| IL-7 | 0.29 (-0.42, 1.01) | 0.59 (-0.12, 1.31) | 0.19 (-0.53, 0.90) |
| IL-8 | -0.06 (-0.78, 0.65) | -1.68 (-2.40, -0.96) | -2.26 (-2.97, -1.54) |
| IL-10 | 0.09 (-0.62, 0.81) | -0.14 (-0.86, 0.57) | -0.21 (-0.93, 0.50) |
| IL-18 | -0.10 (-0.82, 0.61) | 0.55 (-0.17, 1.26) | 2.09 (1.37, 2.80) |
| IL-10RA[a] | -0.10 (-0.68, 0.57) | -0.48 (-0.85, 0.21) | -0.52 (-0.86, 0.17) |
| IL-10RB | -0.35 (-1.06, 0.37) | 0.83 (0.12, 1.55) | 1.01 (0.30, 1.73) |
| IL-12B | -0.14 (-0.85, 0.58) | -0.19 (-0.91, 0.52) | -0.07 (-0.79, 0.65) |
| IL-15RA | -0.81 (-1.52, -0.09) | -1.88 (-2.60, -1.17) | -1.69 (-2.41, -0.98) |
| IL-17C | 0.59 (-0.13, 1.30) | -0.68 (-1.40, 0.03) | -2.42 (-3.13, -1.70) |
| IL-18R1 | -0.55 (-1.27, 0.16) | 0.11 (-0.61, 0.82) | 1.19 (0.47, 1.91) |
| LAP TGF -beta-1 | -1.15 (-1.87, -0.44) | -0.79 (-1.50, -0.07) | 0.42 (-0.29, 1.14) |
| LIF-R | -0.56 (-1.28, 0.15) | -3.05 (-3.76, -2.33) | -2.61 (-3.33, -1.90) |
| MCP-1[a] | -0.26 (-0.76, 0.44) | -0.90 (-0.98, -0.63) | -0.90 (-0.98, -0.63) |

| | | | |
|---|---|---|---|
| MCP-2 | -0.04 (-0.75, 0.68) | 2.79 (2.08, 3.51) | 3.22 (2.50, 3.93) |
| MCP-3 | 0.11 (-0.60, 0.83) | 0.51 (-0.20, 1.23) | 0.49 (-0.22, 1.21) |
| MCP-4 | -0.17 (-0.88, 0.55) | 0.91 (0.19, 1.62) | 1.00 (0.28, 1.71) |
| MMP-1[a] | -0.10 (-0.68, 0.57) | -0.74 (-0.93, -0.21) | -0.74 (-0.93, -0.21) |
| MMP-10[a] | -0.45 (-0.84, 0.25) | 0.00 (-0.63, 0.63) | -0.39 (-0.82, 0.32) |
| NT-3 | -0.79 (-1.50, -0.07) | -4.93 (-5.65, -4.22) | -3.75 (-4.47, -3.04) |
| OPG | -0.61 (-1.33, 0.11) | -2.50 (-3.21, -1.78) | -2.90 (-3.61, -2.18) |
| OSM | -0.26 (-0.98, 0.45) | -1.17 (-1.89, -0.46) | -1.82 (-2.54, -1.11) |
| PD-L1 | -0.65 (-1.36, 0.07) | -2.19 (-2.90, -1.47) | -1.26 (-1.97, -0.54) |
| 4E-BP1 | 0.06 (-0.65, 0.78) | 0.28 (-0.44, 0.99) | 0.11 (-0.61, 0.82) |
| SCF | -0.08 (-0.79, 0.64) | -0.19 (-0.91, 0.53) | -0.57 (-1.28, 0.15) |
| SIRT2 | 0.03 (-0.69, 0.74) | -1.17 (-1.89, -0.46) | -0.87 (-1.58, -0.15) |
| SLAMF1 | -0.34 (-1.06, 0.38) | -0.48 (-1.19, 0.24) | 0.00 (-0.72, 0.71) |
| ST1A1 | 0.01 (-0.70, 0.73) | -0.73 (-1.45, -0.02) | -0.94 (-1.66, -0.23) |
| STAMBP | -0.04 (-0.76, 0.67) | -3.40 (-4.11, -2.68) | -3.24 (-3.95, -2.52) |
| TGFalpha | -0.07 (-0.79, 0.64) | 0.75 (0.03, 1.46) | 0.48 (-0.23, 1.20) |
| TNF | 0.35 (-0.37, 1.06) | -3.33 (-4.05, -2.61) | -4.32 (-5.03, -3.60) |
| TNFB | -0.67 (-1.38, 0.05) | -2.11 (-2.83, -1.40) | -1.07 (-1.78, -0.35) |
| TNFRSF9 | -0.83 (-1.54, -0.11) | -5.78 (-6.50, -5.07) | -3.22 (-3.93, -2.50) |

| | | | |
|---|---|---|---|
| TNFSF14[a] | -0.32 (-0.79, 0.39) | -0.87 (-0.97, -0.53) | -0.90 (-0.98, -0.63) |
| TRAIL | -0.28 (-0.99, 0.44) | -2.99 (-3.70, -2.27) | -4.93 (-5.64, -4.21) |
| TRANCE | -0.14 (-0.85, 0.58) | -1.29 (-2.01, -0.58) | -0.49 (-1.21, 0.23) |
| TWEAK | -0.74 (-1.46, -0.03) | -4.27 (-4.98, -3.55) | -2.55 (-3.27, -1.84) |
| uPA | 0.00 (-0.71, 0.72) | -0.86 (-1.58, -0.15) | -1.10 (-1.81, -0.38) |
| VEGF-A | -0.50 (-1.21, 0.22) | -0.40 (-1.11, 0.32) | -0.09 (-0.81, 0.62) |

[a] *Analyzed through non-parametric tests.* Abbreviations: 4E-BP1, eukaryotic translation initiation factor 4E-binding protein 1; ADA, adenosine deaminase; AXIN1, axin-1; BMI, body mass index; CASP-8, caspase-8; CCL3, C-C motif chemokine 3; CCL4, C-C motif chemokine 4; CCL11, eotaxin; CCL19, C-C motif chemokine 19; CCL20, C-C motif chemokine 20; CCL23, C-C motif chemokine 23; CCL25, C-C motif chemokine 25; CCL28, C-C motif chemokine 28; CD5, T-cell surface glycoprotein CD5; CD6, T cell surface glycoprotein CD6 isoform; CD40, CD40L receptor; CD244, natural killer cell receptor 2B4; CD8A, T-cell surface glycoprotein; CDCP1, CUB domain-containing protein 1; CSF-1, macrophage colony-stimulating factor 1; CST5, cystatin D; CX3CL1, fractalkine; CXCL1, C-X-C motif chemokine 1; CXCL5, C-X-C motif chemokine 5; CXCL6, C-X-C motif chemokine 6; CXCL9, C-X-C motif chemokine 9; CXCL10, C-X-C motif chemokine 10; CXCL11, C-X-C motif chemokine 11; DASH, disabilities of the Arm, Shoulder, and Hand; DNER, delta and Notch-like epidermal growth factor-related receptor; EN-RAGE, Protein S100-A12; FACT-B+4, Functional Assessment of Cancer Therapy-Breast plus 4; FFT, Fast fournier transformation ; FGF-19, fibroblast growth factor 19; FGF-21, fibroblast growth factor 21; FGF-23, fibroblast growth factor 23; Flt3L, fms-related tyrosine kinase 3 ligand; GDNF, glial cell line-derived neurotrophic factor; HF, high Frequency; HGF, hepatocyte growth factor; IFN-gamma, Interferon gamma; IL-6, interleukin 6; IL-7, interleukin-7; IL-8, interleukin 8; IL10, interleukin-10; IL-18, interleukin 18;  IL-10RA, interleukin-10 receptor subunit alpha; IL-10RB, interleukin-10 receptor subunit beta; IL-12B, interleukin-12 subunit beta; IL-15RA, interleukin-15 receptor subunit alpha; IL-17C, interleukin-17C; IL-18R1, interleukin-18 receptor 1; IL-20RA, interleukin 20 receptor subunit alpha; LAP TGF-beta – 1, transforming growth factor beta-1 proprotein; LF, low Frequency; LIF-R, leukemia inhibitory factor receptor; MVPA, moderate-to-vigorous physical activity; MCP-1, monocyte chemotactic protein 1; MCP-2, monocyte chemotactic protein 2; MCP-3, monocyte chemotactic protein 3; MCP-4, monocyte chemotactic protein 4; MMP-1, matrix metalloproteinase-1; MMP-10, matrix metalloproteinase-10; NRS, numerical Rating Scale; NT-3, neurotrophin-3; OSM, oncostatin-M; OPG, osteoprotegerin; PD-L1, programmed cell death 1 ligand 1; RMSSD, Root mean square of the successive differences; RR, intervals between R-wave peaks; SCF, stem cell factor; SDNN, standard deviation of NN (Normal-to-Normal) intervals; SLAMF1, signaling lymphocytic activation molecule; SIRT2, SIR2-like protein 2; STAMBP, STAM-binding protein; ST1A1, sulfotransferase 1A1; TGF-alpha, transforming growth factor alpha; TNF, tumor necrosis factor; TNFB, TNF-beta; TNFRSF9, tumor necrosis factor receptor superfamily member 9; TNFSF14, tumor necrosis factor ligand superfamily member 14; TRAIL, TNF-related apoptosis-inducing ligand; TRANCE, TNF-related activation-induced cytokine; TWEAK, tumor necrosis factor (Ligand) superfamily member 12; uPA, urokinase-type plasminogen activator; VEGF-A, vascular endothelial growth factor A, long form

**Supplementary file S7.** Correlations between outcomes.

**Table S7.1.** Pearson correlation analysis between changes (%Δ) in outcomes that showed a significant time effect and a significant change at post-intervention compared to baseline (based on post-hoc analysis): Affected and unaffected arm-related outcomes and quality of life (n = 11 breast cancer survivors)

| **Affected arm** | | | | | | | | | | | | | | |
|---|---|---|---|---|---|---|---|---|---|---|---|---|---|---|
| **Outcomes correlated** | **Chest press** | | **Row** | | **Handgrip** | | **Pain (*affected arm*)** | | **FACT-B+4 Emotional well-being** | | **FACT-B Total** | | **FACT-B+4 Total** | |
| | Pearson's r | *p-value* | Pearson's r | *p-value* | Pearson's r | *p-value* | Pearson's r | *p-value* | Pearson's r | *p-value* | Pearson's r | *p-value* | Pearson's r | *p-value* |
| **Chest press** | - | - | 0.915 | ***<0.001*** | 0.039 | *0.908* | 0.09 | *0.792* | 0.425 | *0.193* | -0.242 | 0.474 | 0.265 | *0.431* |
| **Row** | 0.915 | ***<0.001*** | - | - | -0.022 | *0.949* | 0.045 | *0.895* | 0.309 | *0.356* | -0.284 | 0.397 | 0.282 | *0.401* |
| **Handgrip** | 0.039 | *0.908* | -0.022 | *0.949* | - | - | 0.530 | *0.094* | 0.147 | *0.667* | 0.246 | 0.465 | 0.205 | *0.545* |
| **Pain** | 0.090 | *0.792* | 0.045 | *0.895* | -0.530 | *0.094* | - | - | 0.127 | *0.710* | -0.576 | 0.064 | 0.566 | *0.069* |

| **Unaffected arm** | | | | | | | | | | | | |
|---|---|---|---|---|---|---|---|---|---|---|---|---|
| | **Chest press** | | **Row** | | **Pain (*affected arm*)** | | **FACT-B+4 Emotional well-being** | | **FACT-B Total** | | **FACT-B+4 Total** | |
| | Pearson's r | *p-value* | Pearson's r | *p-value* | Pearson's r | *p-value* | Pearson's r | *p-value* | Pearson's r | *p-value* | Pearson's r | *p-value* |
| **Chest press** | - | - | 0.656 | ***0.028*** | -0.148 | *0.665* | 0.522 | *0.099* | -0.006 | 0.986 | 0.019 | *0.955* |
| **Row** | 0.656 | ***0.028*** | - | - | -0.139 | *0.684* | 0.125 | *0.713* | -0.001 | 0.999 | 0.033 | *0.923* |

Abbreviations. FACT-B+4, The Functional Assessment of Cancer Therapy - Breast.

*Bold numbers show statistically significant differences: p<0.05.*

**Table S7.2.** Pearson correlation analysis between changes (%Δ) in outcomes that showed a significant time effect and a significant change at post-intervention compared to baseline (based on post-hoc analysis): Quality of life-related outcomes (n = 11 breast cancer survivors)

| **Quality of life** | | | | | | |
|---|---|---|---|---|---|---|
| **Outcomes correlated** | **FACT-B+4 Emotional well-being** | | **FACT-B Total** | | **FACT-B+4 Total** | |
| | Pearson's r | *p-value* | Pearson's r | *p-value* | Pearson's r | *p-value* |
| **FACT-B+4 Emotional well-being** | - | - | 0.377 | *0.254* | 0.458 | *0.156* |
| **FACT-B Total** | 0.377 | *0.254* | - | - | 0.937 | ***<0.001*** |
| **FACT-B+4 Total** | 0.458 | *0.156* | 0.937 | ***<0.001*** | - | - |

Abbreviations. FACT-B+4, The Functional Assessment of Cancer Therapy - Breast.

*Bold numbers show statistically significant differences: p<0.05.*

**Table S7.3.** Pearson correlation analysis between changes (%Δ) in outcomes that showed a significant time effect and a significant change at follow-up compared to baseline (based on post-hoc analysis): Molecular and functional outcomes (n = 10 breast cancer survivors)

| **Outcomes correlated** | Affected arm row | | Unaffected arm row | | 10 cm below elbow – medial (subcutaneous thickness -cm) | |
|---|---|---|---|---|---|---|
| | Pearson's r | *p-value* | Pearson's r | *p-value* | Pearson's r | *p-value* |
| AXIN1 | -0.333 | *0.347* | -0.404 | *0.246* | *0.175* | *0.629* |
| CASP-8 | -0.081 | *0.825* | 0.172 | *0.634* | 0.131 | *0.719* |
| CCL3 | 0.716 | ***0.020*** | 0.582 | *0.077* | 0.687 | ***0.028*** |
| CCL4 | 0.574 | *0.082* | 0.380 | *0.278* | 0.395 | *0.259* |
| CCL11 | 0.779 | ***0.008*** | 0.440 | *0.203* | 0.247 | *0.492* |
| CCL28 | 0.250 | *0.486* | -0.065 | *0.858* | -0.353 | *0.317* |
| CD6 | 0.538 | *0.109* | 0.391 | *0.264* | 0.541 | *0.380* |
| CD40 | 0.222 | *0.538* | 0.255 | *0.478* | 0.312 | *0.380* |
| C3XCL1 | -0.039 | *0.915* | 0.150 | *0.679* | -0.369 | *0.294* |
| CDCP1 | 0.515 | *0.128* | 0.410 | *0.239* | -0.152 | *0.676* |
| CSF-1 | 0.001 | *0.030* | -0.233 | *0.516* | 0.681 | ***0.030*** |
| DNER | 0.156 | *0.668* | 0.226 | *0.530* | -0.701 | ***0.024*** |
| EN-RAGE | -0.441 | *0.202* | -0.255 | *0.477* | -0.070 | *0.847* |
| LIF-R | 0.130 | *0.721* | -0.293 | *0.411* | 0.289 | *0.418* |
| MCP-2 | 0.460 | *0.181* | 0.284 | *0.427* | 0.658 | ***0.039*** |

| | | | | | | |
|---|---|---|---|---|---|---|
| NT-3 | -0.050 | *0.890* | -0.197 | *0.586* | -0.241 | *0.502* |
| OPG | 0.336 | *0.342* | 0.583 | *0.077* | 0.439 | *0.204* |
| PD-L1 | -0.208 | *0.564* | -0.220 | *0.540* | -0.123 | *0.734* |
| STAMBP | 0.562 | *0.091* | 0.517 | *0.126* | 0.204 | *0.573* |
| TNF | -0.107 | *0.768* | -0.300 | *0.400* | -0.384 | *0.274* |
| TNFB | -0.350 | *0.322* | -0.327 | *0.357* | -0.010 | *0.977* |
| TNFRSF9 | -0.128 | *0.725* | -0.298 | *0.403* | -0.550 | *0.100* |
| TRAIL | 0.276 | *0.440* | 0.344 | *0.330* | -0.001 | *0.997* |
| TWEAK | 0.206 | *0.568* | 0.087 | *0.810* | 0.454 | *0.188* |

*Bold numbers show statistically significant differences: $p<0.05$.*

Abbreviations. AXIN1, axin-1; BMI, body mass index; CASP-8, caspase-8; CCL3, C-C motif chemokine 3; CCL4, C-C motif chemokine 4; CCL11, Eotaxin; CCL28, C-C motif chemokine 28; CD6, T cell surface glycoprotein CD6 isoform; CD40, CD40L receptor; CDCP1, CUB domain-containing protein 1; CSF-1, macrophage colony-stimulating factor 1; DNER, delta and Notch-like epidermal growth factor-related receptor; EN-RAGE, protein S100-A12; IL-8, Interleukin 8; LIF-R, leukemia inhibitory factor receptor; MCP-2, monocyte chemotactic protein 2; NT-3, neurotrophin-3; OPG, osteoprotegerin; STAMBP, STAM-binding protein; TNF, tumor necrosis factor; TNFB, TNF-beta; TNFRSF9, tumor necrosis factor receptor superfamily member 9; TRAIL, TNF-related apoptosis-inducing ligand; TWEAK, tumor necrosis factor (Ligand) superfamily, member 12.
